# Multi‐omics integration and machine learning identify an ACKR1^+^ endothelial cell‐based lactate‐immune signature associated with carotid plaque vulnerability and progression

**DOI:** 10.1002/ctm2.70856

**Published:** 2026-10-14

**Authors:** Ji Zhang, Qijian Zhao, Yufei Wu, Keqiang Shu, Junye Chen, Kang Li, Yuyao Feng, Xu Zhang, Wenjian Liu, Zhichao Lai, Bao Liu

**Affiliations:** ^1^ Department of Vascular Surgery Peking Union Medical College Hospital, Chinese Academy of Medical Sciences and Peking Union Medical College Beijing China; ^2^ Faculty of Data Science City University of Macau Macau China; ^3^ Institute of Aging and Health Chinese Academy of Medical Sciences Beijing China; ^4^ School of Aging and Health Peking Union Medical College Beijing China

**Keywords:** ACKR1, atherosclerosis, lactate metabolism, machine learning, vulnerable plaque

## Abstract

**Background:**

Early identification of vulnerable carotid plaques is crucial for stroke prevention, but the upstream metabolic processes linked to endothelial transition and immune recruitment remain poorly defined. We characterised lactate metabolism in ACKR1^+^ endothelial cells (ECs) in human carotid plaques and derived a machine‐learning signature for discriminating plaque vulnerability.

**Methods:**

Internal and external (GSE253903) single‐cell datasets were integrated to identify endothelial subpopulations, and bulk transcriptomic (GSE163154) and in‐house proteomic data were used to assess metabolism–immune correlations. Least absolute shrinkage and selection operator (LASSO) regression selected core features for a logistic model, which was locked and applied to three independent cohorts: GSE43292 (Stary stage ≥ IV vs. I–II), GSE28829 (early vs. advanced lesions) and GSE100927 (normal vs. atherosclerotic arteries). Key molecules were validated in situ by immunohistochemistry (IHC) and spatial transcriptomics in independent plaques (five stable, five unstable).

**Results:**

An ACKR1^+^ EC subpopulation linked to neovascularisation showed hyperactive glycolysis and enhanced lactate metabolism. Computational inference linked lactate accumulation to HIF‐1α stability, CXCL12 release and CD8^+^ T‐cell infiltration via the CXCL12–CXCR4 axis, and the monocarboxylate transporters *SLC16A3* and *SLC16A4* emerged as quantitative readouts of this ‘lactate–immune’ state. In unstable plaques, IHC confirmed higher atypical chemokine receptor 1 (ACKR1; geometric mean ratio [GMR] = 6.82), CD8^+^ T‐cell density (GMR = 4.63), SLC16A3 (GMR = 4.74) and HIF‐1α (GMR = 4.02; all *q* < .05), with lactylation elevated in the lipid core. A four‐feature model (*ACKR1*, *SLC16A3*, *SLC16A4*, CD8^+^ T‐cell cytotoxicity score) performed well in training and retained discrimination in the progression‐stratified cohorts, while in GSE100927 it showed high disease‐identification performance across three arterial beds.

**Conclusions:**

Lactate metabolic reprogramming under hypoxia is associated with ACKR1^+^ EC‐related CD8^+^ T‐cell recruitment and plaque instability. This chain is an association‐based hypothesis requiring functional confirmation. The signature discriminates robustly at both the vulnerability‐stratification and disease‐identification levels, providing a quantifiable framework for molecular classification of atherosclerotic plaques.

## Highlights

- ACKR1^+^ plaque endothelial cells show hyperactive glycolysis and lactate metabolism.
- Lactate is linked to HIF‐1α, CXCL12 and CD8^+^ T‐cell recruitment via CXCL12‐CXCR4.
- SLC16A3 and SLC16A4 are quantitative readouts of this lactateimmune state.
- IHC shows higher ACKR1, SLC16A3, HIF‐1α and CD8^+^ T cells in unstable plaques.
- A four‐feature signature discriminates plaque vulnerability and identifies disease.

## Graphical abstract

ACKR1^+^ endothelial cells mark intraplaque neovessels with hyperactive glycolysis and lactate metabolism. Lactate, HIF‐1α and CXCL12 link this state to CXCR4^+^ CD8^+^ T‐cell recruitment in situ. *SLC16A3* and *SLC16A4* serve as quantitative readouts of this lactate–immune state. A four‐feature signature discriminates lesion progression and identifies lesions across three arterial beds.

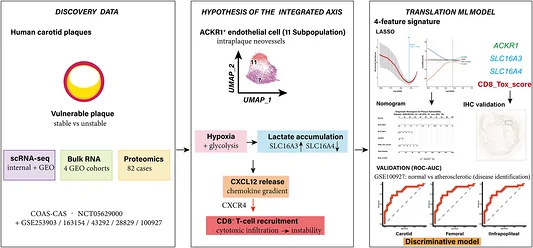

## 1 INTRODUCTION

The rupture of carotid atherosclerotic plaques is the primary cause of ischaemic stroke and fatal cardiovascular events.^1^ In clinical practice, the early identification of ‘vulnerable plaques’ with a high risk of rupture is crucial for preventing acute cerebrovascular events. Although current imaging technologies have made considerable progress in evaluating plaque morphology, it remains challenging to non‐invasively capture the deep molecular biological processes occurring within the plaque. In recent years, with the deep integration of artificial intelligence into medicine, machine‐learning (ML) models based on multi‐omics data have emerged as a cutting‐edge approach to overcoming this bottleneck, as they can translate high‐dimensional molecular transcriptomic data into quantifiable molecular discriminant scores.^2^, ^3^ However, existing predictive models predominantly rely on broad pools of inflammatory or lipid metabolism genes and lack a precise focus on specific pathogenic microenvironments and intercellular communication, which somewhat limits their biological interpretability and potential for clinical translation.

Targeting the immune microenvironment has been recognised as a promising therapeutic strategy for stabilising vulnerable plaques.^4^ In the search for more specific predictive targets, ‘immunometabolic’ reprogramming within the plaque microenvironment has attracted our attention. Immunometabolic reprogramming refers to adaptive changes in energy metabolic pathways (e.g., a shift from oxidative phosphorylation [OXPHOS] to aerobic glycolysis) that occur in intraplaque immune and stromal cells under the influence of local hypoxia, lipid accumulation and chronic inflammation; this metabolic state, in turn, regulates their immune phenotypes and effector functions. Recent studies have shown that, within the plaque microenvironment, the energy metabolic state of immune cells changes with their activation status and, in turn, influences lesion progression.^5^, ^6^ More importantly, metabolites themselves can act as signalling molecules. Lactate, for example, is not only the end product of glycolysis but can also directly regulate gene expression through histone and non‐histone lactylation, an epigenetic modification, thereby tightly coupling the ‘metabolic state’ and ‘immune function’ of cells.^7^ This concept linking metabolism and immunity provides a new perspective for identifying more specific predictive targets within the plaque microenvironment.

Single‐cell analysis has played an increasingly important role in unravelling the tissue heterogeneity of atherosclerosis and advancing precision medicine.^8^ Single‐cell RNA sequencing (scRNA‐seq) studies have confirmed that T cells, particularly CD8^+^ T cells, dominate human atherosclerotic plaques, and their extensive infiltration is closely associated with plaque instability.^9^ A recent breakthrough study published in *Circulation* further elucidated this process using a three‐dimensional human atherosclerotic plaque culture model: intraplaque vascular endothelial cells (ECs) highly expressing atypical chemokine receptor 1 (ACKR1) can potently recruit CD8^+^ T cells by secreting C‐X‐C motif chemokine ligand 12 (CXCL12).^10^ However, that study did not further clarify the association between the recruitment process mediated by this specific cell type and plaque instability. Concurrently, high glycolytic activity and lactate accumulation induced by the hypoxic plaque microenvironment are being redefined from mere metabolic waste to core epigenetic regulators. Several authoritative cardiovascular studies published in 2025 have highlighted that lactate‐mediated ‘lactylation’ plays a critical ‘double‐edged sword’ role in inflammation and angiogenesis in cardiovascular diseases.^11^ Notably, lactate‐induced lactylation of hypoxia‐inducible factor 1α (HIF‐1α) can markedly enhance HIF‐1α protein stability by blocking its recognition by the von Hippel–Lindau (VHL) ubiquitination complex.^12^ Building on these advances, we proposed a potential pathogenic axis underlying plaque vulnerability: lactate accumulated in the hypoxic microenvironment may drive the pro‐inflammatory transition of ACKR1^+^ ECs by stabilising HIF‐1α, thereby triggering the pathogenic infiltration of CD8^+^ T cells.

Although recent bioinformatic studies have begun to focus on macroscopic transcriptomic aberrations of lactylation‐related genes in atherosclerosis,^13^ no study to date has integrated ‘lactate metabolism’ with the immune recruitment function of a specific single‐cell subpopulation (ACKR1^+^ ECs), and ML‐based predictive tools developed on the basis of this intricate mechanistic network are lacking.

To bridge the gap between biological mechanisms and molecular classification, this study adopted a strategy of ‘computation‐driven mechanistic inference and model development’. We first integrated scRNA‐seq and bulk RNA sequencing (bulk RNA‐seq) data to verify, at the multidimensional transcriptomic level, the existence of a ‘lactate–immune’ crosstalk network centred on ACKR1^+^ ECs. More importantly, using this mechanistic network as a feature pool, we applied the least absolute shrinkage and selection operator (LASSO) penalised regression algorithm for rigorous high‐dimensional feature reduction and, in combination with leave‐one‐out cross‐validation (LOOCV), constructed an ML‐based four‐feature lactate–immune risk score model. Through blind validation across multiple independent external cohorts encompassing different sequencing platforms and vascular beds (primarily carotid arteries, together with some femoral and infrapopliteal arteries), this study aims to provide a rigorously statistically validated tool for the molecular classification of plaque vulnerability that combines discriminative performance with biological interpretability.

## 2 MATERIALS AND METHODS

### 2.1 Multi‐omics data acquisition and pre‐processing

#### 2.1.1 Source and description of in‐house scRNA‐seq and proteomic data

To profile the plaque microenvironment at the single‐cell level, we included scRNA‐seq data from 10 in‐house carotid atherosclerotic plaque samples, comprising seven stable and three unstable plaques. Concurrently, we extracted proteomic data from 82 in‐house carotid plaques (grouped into stable and unstable phenotypes) for cross‐validation of metabolic pathways, including 37 stable and 45 unstable plaques. Detailed descriptions of the in‐house scRNA‐seq and proteomic data can also be found in our recently published studies.^14^, ^15^ The 82‐case proteomic dataset was originally generated as part of our previous work in this group and is re‐analysed here within an independent multi‐omics framework addressing a distinct scientific question.

*Pathological assessment of plaque stability*. Plaques in the in‐house cohort were classified as stable or unstable primarily on the basis of haematoxylin–eosin‐stained sections, supplemented by Masson trichrome and CD68 staining. Two vascular pathologists blinded to clinical information independently evaluated the sections according to the American Heart Association (AHA) histological classification of atherosclerotic lesions,^16^ the modified Virmani classification,^17^ and the Oxford Plaque Study histological criteria for carotid plaques.^18^ Unstable (vulnerable) plaques were defined as those exhibiting one or more of the following features: a thin or ruptured fibrous cap (thin‐cap fibroatheroma), a large lipid/necrotic core, dense macrophage/inflammatory cell infiltration, intraplaque haemorrhage (IPH) and surface ulceration or thrombosis (corresponding to AHA type VI complicated lesions). Stable plaques were defined as those with a thick and intact fibrous cap, a small lipid core, abundant smooth muscle cells (SMCs) and collagen, sparse inflammatory cells and no surface disruption or haemorrhage (corresponding to AHA type IV–V fibrous/fibrocalcific plaques). Disagreements between the two evaluators were resolved by consensus or adjudicated by a third senior pathologist.

The Carotid Artery Stenosis Study (COAS‐CAS; ClinicalTrials.gov: NCT05629000) is a prospective study conducted at Peking Union Medical College Hospital (PUMCH), aiming to collect atherosclerotic carotid plaques and associated clinical characteristics from patients undergoing carotid endarterectomy (CEA). The study was approved by the Ethics Review Committee of PUMCH (Approval Nos. JS‐2966 and I‐22PJ019). Written informed consent was obtained from all patients before participation, and all patients consented to the publication of the research data. Between May 2015 and March 2022, we enrolled patients at PUMCH who underwent either standard CEA or eversion CEA (eCEA). The eligibility criteria included asymptomatic carotid stenosis of 70%–99% and symptomatic carotid stenosis of 50%–99%, as defined by the North American Symptomatic Carotid Endarterectomy Trial (NASCET) criteria. Clinical data were systematically collected for each participant upon admission. All enrolled patients underwent preoperative carotid duplex ultrasonography confirming carotid stenosis of at least 50%. This study strictly adhered to the ethical principles of the Declaration of Helsinki.

For scRNA‐seq analysis, fresh tissues were rinsed with phosphate‐buffered saline (PBS), mechanically minced and digested in the dark at 37°C for 1 h in a mixture containing multiple types of collagenase (types II, IV and XI;  .3–1 mg/mL), DNase and hyaluronidase. Digestion was terminated by adding foetal bovine serum. The digested suspension was filtered sequentially through 70‐µm and 40‐µm cell strainers, centrifuged at 300 × *g* for 8 min at 4°C, and subjected to red blood cell lysis. Single cells were finally resuspended at a concentration of 1250 cells/µL. Library construction, including droplet generation, reverse transcription and cDNA amplification, was performed using the DNBelab C Series Single‐Cell Library Prep Set (MGI). After quantification using a Qubit ssDNA Assay Kit, the libraries were sequenced on the DNBSEQ platform using a paired‐end strategy (Read 1: 47 bp; Read 2: 100 bp).

For proteomic analysis, excised carotid plaques were rinsed with physiological saline, fixed in 10% formalin and decalcified in 10% acetic acid. Lesion tissues from the proximal (20 mm) and distal (15 mm) segments centred on the carotid bifurcation were collected. Because calcification is common in carotid plaques, decalcification was a prerequisite for serial sectioning and histopathological classification; samples for mass spectrometry and pathological classification were obtained from the same paraffin block to ensure that the proteomic data corresponded to the tissue region on which group assignment was based. Tissue sections were treated with protein extraction buffer at 100°C for 20 min to reverse formalin‐induced protein cross‐linking and then digested using the filter‐aided sample preparation method. Briefly, the protein solution was reduced with dithiothreitol, alkylated with iodoacetamide in the dark and purified using a 30‐kDa ultrafiltration filter, followed by overnight digestion with trypsin (1:50, w/w) at 37°C. The resulting peptides were desalted using Oasis C18 columns and lyophilised by vacuum centrifugation; all samples were processed using the same standardised workflow, with fixation time and decalcification conditions kept consistent across groups. After reconstitution, peptides were separated on an UltiMate 3000 system equipped with an in‐house packed C18 analytical column (75 µm × 50 cm; column temperature, 60°C) using a 25‐min stepped linear gradient (organic phase from 5% to 50%; flow rate, 1.5 µL/min). Mass spectrometry was performed on an Orbitrap Exploris 480 mass spectrometer in positive data‐independent acquisition (DIA) mode, with the MS1 full‐scan resolution set to 120 000 (*m*/*z* 350–1200) and the MS2 resolution set to 30 000. Raw data were imported into Spectronaut Pulsar (v14) and searched against the human SwissProt database to generate a spectral library (precursor and fragment ion mass tolerances, 10 ppm and  .02 Da, respectively; maximum missed cleavages, 2). Data were filtered at a *Q* value <.01 (1% false discovery rate [FDR]); only proteins detected in >80% of samples within a group were retained, and missing values were imputed using the k‐nearest neighbour method.

#### 2.1.2 External scRNA‐seq and bulk transcriptomic data

The external scRNA‐seq validation dataset GSE253903 was retrieved from the Gene Expression Omnibus (GEO) database and comprises carotid atherosclerotic plaque samples from six symptomatic and six asymptomatic individuals. Details of sample processing are available in the GSE253903 record on the GEO website.

The GSE163154 dataset was used as the training cohort and includes 43 carotid plaque samples from patients undergoing CEA, categorised into IPH and non‐IPH groups. The samples in this cohort were obtained from symptomatic patients undergoing CEA, and group comparisons were based on IPH status rather than on the presence or absence of symptoms. The GSE43292, GSE28829 and GSE100927 datasets from GEO were used for independent external validation; detailed information on these datasets is available on the GEO website.

GSE43292 comprises paired CEA tissue samples from 32 patients, including atherosclerotic plaque samples from the plaque core and shoulder (Stary stage ≥ IV) and macroscopically intact distant tissue samples (Stary stages I–II). GSE28829 contains carotid atherosclerotic samples comprising 13 early lesions (pathological intimal thickening and intimal xanthoma) and 16 advanced lesions (thin‐ or thick‐cap fibroatheroma). GSE100927 includes 104 atherosclerotic and non‐atherosclerotic control arterial samples from carotid, femoral and infrapopliteal arteries. All expression matrices were obtained using the *GEOquery* R package or downloaded directly from the GEO website. For microarray data, probes were converted to official gene symbols using the corresponding annotation packages (e.g., *illuminaHumanv2.db*), and the mean expression value was retained for genes mapped by multiple probes. All expression matrices underwent background correction, log_2_ transformation and Z‐score standardisation; when the locked‐coefficient model was applied to the external cohorts, Z‐score standardisation was performed independently within each external cohort.

### 2.2 Processing of scRNA‐seq data

#### 2.2.1 Data pre‐processing and quality control

scRNA‐seq data were loaded and initially processed using the *Seurat* R package (v5). To obtain high‐quality single‐cell expression profiles, rigorous quality control was performed for each sample.

For the in‐house single‐cell cohort, cells with 300–6000 detected genes (nFeature_RNA) were retained; this lower limit was specifically optimised to capture resting ECs. Cells with a mitochondrial gene proportion >15% (stressed or dying cells) or an erythrocyte gene proportion >5% (blood contamination) were removed. The external GSE253903 single‐cell cohort was processed using similar criteria: cells with nFeature_RNA of 500–6000 were retained, and the same thresholds for mitochondrial (<15%) and erythrocyte (<5%) gene proportions were applied.

After removal of low‐quality cells, the *DoubletFinder* algorithm (v3) was applied independently to each sample to eliminate interference from droplets containing multiple cells (doublets). According to the 10× Genomics capture efficiency standards, the doublet rate was set at approximately  .8% per 1000 cells to estimate the expected number of doublets (nExp), and only cells identified as ‘singlets’ were retained for downstream analyses.

#### 2.2.2 Dimensionality reduction, integration and clustering

To eliminate technical variation arising from inter‐individual differences or sequencing batches, batch effects were corrected using the *Harmony* R package. After the high‐quality cells within each cohort were merged, the data were log‐normalised (*NormaliseData*), and the top 2000 highly variable features were identified using the variance‐stabilising transformation (vst) method. The expression matrix was then scaled (*ScaleData*) and subjected to principal component analysis (PCA), and the first 30 principal components were retained for downstream analyses.

Based on the PCA results, the *Harmony* algorithm was run with sample origin (PatientID or GSM number) as the covariate to correct for batch effects. The first 20 Harmony dimensions were then used to construct a cell neighbourhood graph and perform unsupervised clustering with the *FindNeighbours* and *FindClusters* functions (resolution = .6). Finally, uniform manifold approximation and projection (UMAP) was applied for two‐dimensional visualisation.

#### 2.2.3 Cell‐type annotation and subpopulation extraction

Based on the unsupervised clustering results, cell types were manually annotated according to the expression patterns of canonical marker genes reported in established literature.^14^ The major cell types identified are summarised in Table 1.

**TABLE 1:** Marker genes used for annotation of cell types in the internal single‐cell cohort.

Cell type	Gene annotation
CD8^+^ T cells	*‘CD3D’,’CD3E’,’CD8A’,’CD8B’, ‘GZMA’, ‘GZMB’, ‘PRF1’, ‘NKG7’*
T_cells	*‘CD3D’, ‘CD3E’, ‘CD8A’, ‘CD8B’*
NKT_cells	*‘CD3D’, ‘CD3E’, ‘CD8A’, ‘CD8B’, ‘GNLY’, ‘NKG7’, ‘PRF1’*
Monocytes	*‘FCGR3A’, ‘FCN1’*
Macrophages	*‘CD68’, ‘MSR1’, ‘APOE’, ‘C1QA’*
cDC1	*‘XCR1’, ‘CLEC9A’*
cDC2	*‘CD1C’, ‘CLEC10A’, ‘FCER1A’*
pDCs	*‘LILRA4’, ‘SPIB’, ‘IRF7’*
Endothelial (ECs)	*‘PECAM1’, ‘CDH5’, ‘VWF’*
Arterial ECs (ACKR1^−^)	*‘GJA4’, ‘GJA5’, ‘GATA2’, ‘MECOM’*
Venous/neovessel ECs (ACKR1^+^)	*‘ACKR1’, ‘NR2F2’, ‘PLVAP’, ‘CXCL12’*
Smooth muscle cells (SMCs)	*‘COL1A2’, ‘COL3A1’, ‘ACTA2’, ‘MYH11’*
B_cells	*‘CD79A’, ‘CD79B’, ‘MS4A1’*
Plasma_cells	*‘JCHAIN’, ‘IGHG1’, ‘IGHA1’*
Mast_cells	*‘KIT’, ‘CPA3’*
Proliferative	*‘MKI67’*

*Note*: Clusters were defined by unsupervised clustering and assigned to cell types using the markers listed.

After global cell annotation, EC subpopulations expressing distinct markers (e.g., ACKR1^+^ ECs and ACKR1^−^ ECs) were extracted and saved as independent objects for targeted downstream mechanistic analyses. Of note, the partitioning of EC subpopulations was driven by unsupervised clustering rather than by an arbitrary threshold of *ACKR1* expression. After global annotation, ECs (positive for *PECAM1*, *CDH5* and *VWF*) naturally separated into two transcriptionally distinct clusters (Clusters 11 and 7 in the in‐house cohort; Clusters 7 and 10 in the external GSE253903 cohort). Subpopulations were extracted strictly according to cluster identity (seurat_clusters), prior to and independently of any observation of *ACKR1* expression. In the differential expression analysis between the two clusters, *ACKR1* was significantly and specifically enriched in one cluster (in‐house cohort: average log_2_ fold change [avg log_2_FC] = 2.37, adjusted *p* = 8.57e−222; proportion of positive cells, 51.1% vs. 13.5%), which was therefore annotated as ACKR1^+^ ECs, whereas the other cluster was annotated as ACKR1^−^ ECs. Here, ‘+/−’ denotes relative enrichment between two transcriptional states rather than absolute presence or absence; that is, ACKR1^−^ ECs do not completely lack *ACKR1* expression, and their low‐level signal reflects the basal expression and technical background commonly observed in single‐cell data. Because clustering is based on the overall transcriptional similarity of thousands of genes rather than a hard cut‐off of a single gene, this classification is insensitive to background expression in individual cells and does not compromise the biological reliability of the downstream differential expression, gene set enrichment analysis (GSEA) or transcription factor (TF) activity analyses. The distribution of *ACKR1* expression in the two subpopulations is shown in Supporting Information Figure 5A,B.

#### 2.2.4 Differentially expressed genes and pathway enrichment analysis

For the extracted EC subpopulations, differentially expressed genes (DEGs) were identified using the *FindMarkers* function in *Seurat* (Wilcoxon rank‐sum test). Genes with an adjusted *p* value (*p*_val_adj) <.05 and |avg log_2_FC| >.5 were defined as significant DEGs.

Gene Ontology biological process (GO‐BP) and Kyoto Encyclopedia of Genes and Genomes (KEGG) pathway enrichment analyses of significantly upregulated and downregulated gene sets were performed using the *clusterProfiler* R package. Furthermore, to comprehensively evaluate transcriptional trends, all background genes were ranked without log_2_FC threshold filtering, and GSEA was performed using the Molecular Signatures Database (MSigDB) HALLMARK gene sets retrieved via the *msigdbr* package to assess the activation or inhibition of core biological pathways such as hypoxia and glycolysis.

#### 2.2.5 Transcription factor activity inference

To infer upstream TF activity changes within EC subpopulations, the DoRothEA database was used in combination with the VIPER algorithm. High‐confidence human regulons (confidence levels A, B and C) were extracted from DoRothEA, and TF activity scores were inferred from the single‐cell gene expression matrix. Based on differences in activity scores between subpopulations, key TFs with significant activation or inhibition (e.g., *p*_val_adj <.05) were identified and visualised.

#### 2.2.6 Metabolic signature scoring

To quantitatively assess metabolic remodelling at the single‐cell level, module scores were calculated using the *AddModuleScore* function in *Seurat*. The evaluated gene sets included the HALLMARK OXPHOS gene set and a lactylation‐ and metabolism‐related gene set (lactylation signature) integrated from the literature, which includes core metabolic and modification enzymes such as *LDHA*, *LDHB*, *SLC16A1*, *SLC16A3* and *EP300*.^19^ In addition to the OXPHOS and lactylation modules, glycolytic activity was assessed using two independent scores calculated with identical *AddModuleScore* parameters: one based on the broad HALLMARK_GLYCOLYSIS gene set (198 genes detected in the present dataset) and the other based on a targeted gene set of rate‐limiting glycolytic enzymes (*HK2*, *PFKL*, *PFKM*, *PFKP*, *PFKFB3*, *ALDOA*, *GAPDH*, *PKM*, *LDHA*, etc.). Differences in scores between subpopulations were evaluated using the Wilcoxon rank‐sum test. Because single‐cell analyses may include thousands of cells, in which even minimal between‐group differences can yield extremely small *p* values, Cliff's *δ* was also reported as an effect size, and its magnitude was interpreted according to the thresholds proposed by Romano et al. (|*δ*| < .147, negligible;  .147–.33, small;  .33–.474, medium; ≥.474, large) to avoid overestimating the actual magnitude of between‐group differences on the basis of *p* values alone.^20^

#### 2.2.7 Cell–cell communication analysis

To evaluate ligand–receptor communication between ACKR1^+^ ECs and CD8^+^ T cells, cell–cell communication in the in‐house single‐cell data was inferred using the *CellChat* R package (v1.6.1). A CellChat object was constructed from the LogNormalise‐normalised expression matrix of the Seurat object (RNA assay, data layer) and the cell‐type annotations. During annotation, cells of the same type distributed across multiple clusters were merged into unified populations (T cells: Clusters 0/4/10/15; macrophages: Clusters 3/5; SMCs: Clusters 2/16) to increase the statistical power of each population, whereas ACKR1^+^ ECs (Cluster 11) and ACKR1^−^ ECs (Cluster 7) were kept separate. Because the CXCL12–C‐X‐C chemokine receptor 4 (CXCR4) pair of interest represents secreted chemotactic signalling, the Secreted Signalling subset of CellChatDB.human was used as the ligand–receptor database. The *subsetData*, *identifyOverExpressedGenes* and *identifyOverExpressedInteractions* functions were executed sequentially; communication probabilities were then computed using *computeCommunProb* (type = ‘triMean’; default, 100 permutations), populations with too few cells were filtered using *filterCommunication* (min.cells = 10), and pathway‐level networks were finally aggregated using *computeCommunProbPathway* and *aggregateNet*.

Given the low detection rates of chemokine receptors in single‐cell data and the potential dilution of sparse signals by highly abundant, broadly expressed pathways, we additionally performed a high‐sensitivity supplementary analysis. In this analysis, T‐cell annotation was refined: Cluster 4, which highly expressed *CXCR4*, was separately labelled as CD8^+^ T cells (C4), and Cluster 15 was labelled as other CD8^+^ T cells, to prevent dilution of the target subpopulation signal by other T cells. In addition, the thresholds for identifying overexpressed genes were relaxed (thresh.pc = .01, thresh.fc = 1.05, thresh.p = .1), the statistic for probability calculation was changed to the truncated mean (type = ‘truncatedMean’, trim = .05), and the minimum number of cells was lowered to min.cells = 5. Under these settings, with ACKR1^+^ ECs as senders and CD8^+^ T cells (C4) as receivers, all significant ligand–receptor pairs and chemokine pathways restricted by signalling = ‘CXCL’ were retrieved.

### 2.3 Identification of ACKR1^+^ ECs and metabolic characterisation

Among the ECs identified in our in‐house scRNA‐seq data, we identified a subpopulation (Cluster 11) characterised by specific high expression of *ACKR1*. With reference to the study by Parma et al.^10^ on intraplaque neovessel‐mediated CD8^+^ T‐cell recruitment, we annotated this subpopulation as ACKR1^+^ ECs and validated its homology in the external GSE253903 dataset. Subsequently, the metabolic pathways of this subpopulation were scored using the *AddModuleScore* function, with a particular focus on glycolysis and lactate metabolism. The analysis revealed that this subpopulation exhibited an elevated glycolysis score accompanied by *HIF1A* overexpression.

### 2.4 Computational construction of the metabolic–immune crosstalk axis

Integrating recent findings that lactylation enhances HIF‐1α stability by blocking VHL recognition,^12^ we formulated a computation‐driven mechanistic hypothesis: lactate generated in the hypoxic and highly glycolytic plaque microenvironment may promote HIF‐1α accumulation, which may in turn be associated with upregulated *CXCL12* expression in ACKR1^+^ ECs and contribute to local CD8^+^ T‐cell recruitment via the CXCL12–CXCR4 axis, thereby being associated with plaque instability.

To validate this hypothesis at the bulk transcriptomic level, we extracted from the GSE163154 cohort the expression profiles of *ACKR1*, *HIF1A*, and core enzymes of glycolysis and lactate metabolism, as well as a CD8^+^ T‐cell cytotoxicity score (CD8_Tox_Score) composed of *CD8A*, *CD8B*, *GZMA*, *GZMB* and *PRF1*.^21^ Pearson correlation analysis was used to evaluate the co‐expression network of these key nodes at the patient transcriptional level. Furthermore, using our in‐house proteomic data, we performed enrichment analysis of significantly upregulated glycolytic proteins in the unstable plaque group to provide omics‐level support for the metabolism‐driven mechanism.

### 2.5 Cell composition adjustment analysis

Because correlations between genes in bulk tissue transcriptomes may partly arise from differences in cell‐type abundance among samples (confounding by cell composition), two supplementary analyses were performed for key molecular associations. First, cell‐type deconvolution and partial correlation analysis: using the average expression profiles of the major cell types (ECs, T cells, macrophages, SMCs, etc.) in the in‐house single‐cell data as references, the cell‐type proportions of each sample in GSE163154 were estimated using non‐negative least squares. Pearson partial correlations between key gene pairs were then calculated while controlling for the proportions of the major cell types and compared with the unadjusted correlation coefficients. Second, pseudo‐bulk analysis: single‐cell data were aggregated into pseudo‐bulk expression profiles by ‘sample × cell type’, and correlation analysis was strictly restricted to a single‐cell population, ACKR1^+^ ECs (Cluster 11), and calculated across samples, thereby excluding, in principle, the influence of differences in cell composition on the associations. Partial correlation analysis and deconvolution were performed using the *ppcor* and *nnls* packages, respectively.

### 2.6 Machine learning‐based feature selection and diagnostic modelling

#### 2.6.1 Pre‐processing and standardisation of expression data

For the training cohort GSE163154, the normalised expression matrix provided by GEO was used. Probes were mapped to gene symbols using *illuminaHumanv2.db*; when a probe mapped to multiple genes, the first annotation was retained, and when multiple probes mapped to the same gene, their expression values were averaged to obtain gene‐level values. The CD8_Tox_Score was constructed from five genes (*CD8A*, *CD8B*, *GZMA*, *GZMB* and *PRF1*): each gene was first Z‐score standardised across samples, and the mean of the five standardised values was then calculated. Subsequently, the candidate feature matrix was Z‐score standardised by gene (column), so that each feature had a mean of 0 and a standard deviation (SD) of 1 within the training set. LASSO feature selection, penalty parameter selection and logistic model fitting were all performed on this standardised scale, on which the final locked regression coefficients were also defined.

To construct an interpretable model for the molecular classification of plaque vulnerability, we extracted the core metabolic–immune variable set obtained from the cross‐validation of the aforementioned scRNA‐seq and proteomic data. In the training set (GSE163154), LASSO‐penalised logistic regression (LR; *glmnet* R package) was first applied to eliminate collinearity among high‐dimensional variables and perform feature reduction. The optimal penalty parameter (lambda.min) was determined by 10‐fold cross‐validation, and four core features with the highest predictive value were ultimately identified. Based on these four features, a multivariable LR diagnostic model was constructed. To avoid overfitting bias associated with the small sample size, the rigorous LOOCV method (*caret* R package) was applied within the training set to evaluate the internal predictive performance of this core signature.

To avoid data leakage and optimism bias arising from performing feature selection and internal validation in the same cohort, the internal performance of the model was evaluated using fully nested cross‐validation: in each fold of LOOCV, LASSO feature selection and model fitting were performed independently within the training subset, and predictions were made only for the held‐out sample, so that the held‐out sample never participated in feature selection. In addition, the apparent performance was optimism‐corrected using 1000 bootstrap resamples, in which the entire modelling pipeline (including LASSO feature selection) was fully repeated within each bootstrap sample. The internal performance of the model was reported as the apparent area under the receiver operating characteristic curve (AUC), the nested LOOCV AUC and the optimism‐corrected AUC.

Model calibration was assessed using the calibration slope, calibration intercept (calibration‐in‐the‐large) and Brier score. The calibration slope was defined as the coefficient of a LR model fitted with the logit of the predicted probability as the sole independent variable (ideal value, 1), and the calibration intercept was defined as the intercept when this logit was included as an offset (ideal value, 0). Given the limited sample size of the training set (*N* = 43), quasi‐complete separation occurred in 3.9%–14.5% of bootstrap resamples, in which case slope estimates from ordinary LR diverge; therefore, the calibration slope was estimated using Firth penalised regression, and optimism was summarised using the median rather than the mean. In addition to bootstrap correction with the locked features, leakage‐free calibration metrics were derived from the held‐out predictions of fully nested LOOCV.

### 2.7 Within‐patient paired analysis and control of clinical confounding

#### 2.7.1 Within‐patient paired analysis

The external validation cohort GSE43292 has a within‐patient paired design comprising 32 patients. After the locked coefficients from the training set were applied to this cohort without modification, score differences between the plaque side and the ipsilateral intact tissue side were calculated with the patient as the pairing unit and compared using the Wilcoxon signed‐rank test and the paired *t*‐test, and the paired concordance rate was evaluated using the exact binomial test. Conditional LR stratified by patient (*clogit* function in the *survival* package) was used to estimate the odds ratio (OR) per 1‐SD increase in the score. To avoid inflated precision due to the paired structure, cluster bootstrap confidence intervals (CIs) were also calculated with the patient as the resampling unit. The single‐feature analyses of the four model components were corrected using the Benjamini–Hochberg (BH) method. Between‐patient variation was estimated as the intraclass correlation coefficient using a linear mixed‐effects model (score ∼ tissue + (1|patient)).

#### 2.7.2 Adjustment for clinical covariates in the in‐house cohort

Patient‐level clinical variables for the in‐house proteomic cohort (37 stable and 45 unstable plaques) were obtained from the systematic clinical data collected at admission in the COAS‐CAS cohort. Fourteen covariates—age, sex, hypertension, diabetes, smoking, hyperlipidaemia, coronary heart disease, history of cerebral infarction (including lacunar infarction) or transient ischaemic attack (TIA), degree of ipsilateral carotid stenosis, low‐density lipoprotein cholesterol, high‐density lipoprotein cholesterol, triglycerides, high‐sensitivity C‐reactive protein and fasting blood glucose—were incorporated into the *limma* design matrix. Differential protein analysis between stable and unstable plaques was then rerun using a pipeline identical to that of the unadjusted analysis, and effect sizes and significance were compared protein by protein. The degree of ipsilateral stenosis was dichotomised as ‘severe or above’ (the original categories ‘postoperative’ and ‘postoperative restenosis’ were assigned to this category because they reflected previously severe lesions); a sensitivity analysis using the strict 0–3 ordinal grading yielded consistent conclusions. Missing covariate values, which were few, were imputed using the median.

### 2.8 Independent external validation and nomogram construction

#### 2.8.1 Standardisation of external cohorts

Because the model coefficients were defined on a standardised scale, all external validation cohorts (GSE43292, GSE28829 and GSE100927) were processed using the same workflow as the training set: the normalised matrices provided by GEO were used; probes were mapped to gene symbols using the annotation package corresponding to each platform (*hugene10sttranscriptcluster.db* for GSE43292 and GSE100927), and values from multiple probes were averaged. Each gene was then Z‐score standardised independently within each cohort, using the mean and SD of that cohort as the reference rather than the standardisation parameters of the training cohort; if the SD of a gene was zero within a cohort, its standardised value was set to 0. The CD8_Tox_Score was recalculated within each cohort using the same five genes and the same method. After standardisation, the feature values were entered into the regression equation with the locked coefficients from the training set to directly calculate risk scores and predicted probabilities, without coefficient refitting, feature reselection or decision threshold adjustment at any stage. In this study, the ‘risk score’ refers to the continuous discriminant value output by the model, which is used to distinguish plaque samples of different pathological grades, and does not denote the probability of a clinical event in patients.

Within‐cohort standardisation was adopted because the cohorts were derived from different detection platforms, with systematic differences in the scale and distribution of absolute expression values; applying the training set parameters or using raw expression values would introduce cross‐platform scale shifts. Because this standardisation relied solely on the expression values themselves and did not use any outcome information, it did not constitute information leakage.

The feature formula and corresponding weight coefficients established in the training set were subjected to ‘model locking’, without any refitting or parameter adjustment, and directly applied to three completely independent external validation cohorts (GSE43292, GSE28829 and GSE100927). These cohorts encompass different sequencing platforms and plaque progression stages (e.g., early vs. advanced lesions; Stary stages I–II vs. Stary stage ≥ IV; normal vs. atherosclerotic tissues). Samples in these external cohorts were never involved in any step of feature selection, model construction or parameter optimisation; therefore, the validation results represent independent, leakage‐free assessments.

Risk probabilities were calculated for the samples in each cohort, receiver operating characteristic (ROC) curves were plotted, and AUCs were computed; calibration was assessed using calibration curves, the Brier score and the Hosmer–Lemeshow test, and clinical utility was evaluated using decision curve analysis (DCA). To visualise the relative weight of each variable in the model, a nomogram was constructed using the *rms* R package. Of note, this nomogram is intended to present the internal structure of the model rather than to calculate individual patient risk probabilities in clinical practice, because the model input consists of molecular features of ex vivo plaque tissue (see Section 4). The overall study design is illustrated in Figure 1.

**FIGURE 1:**
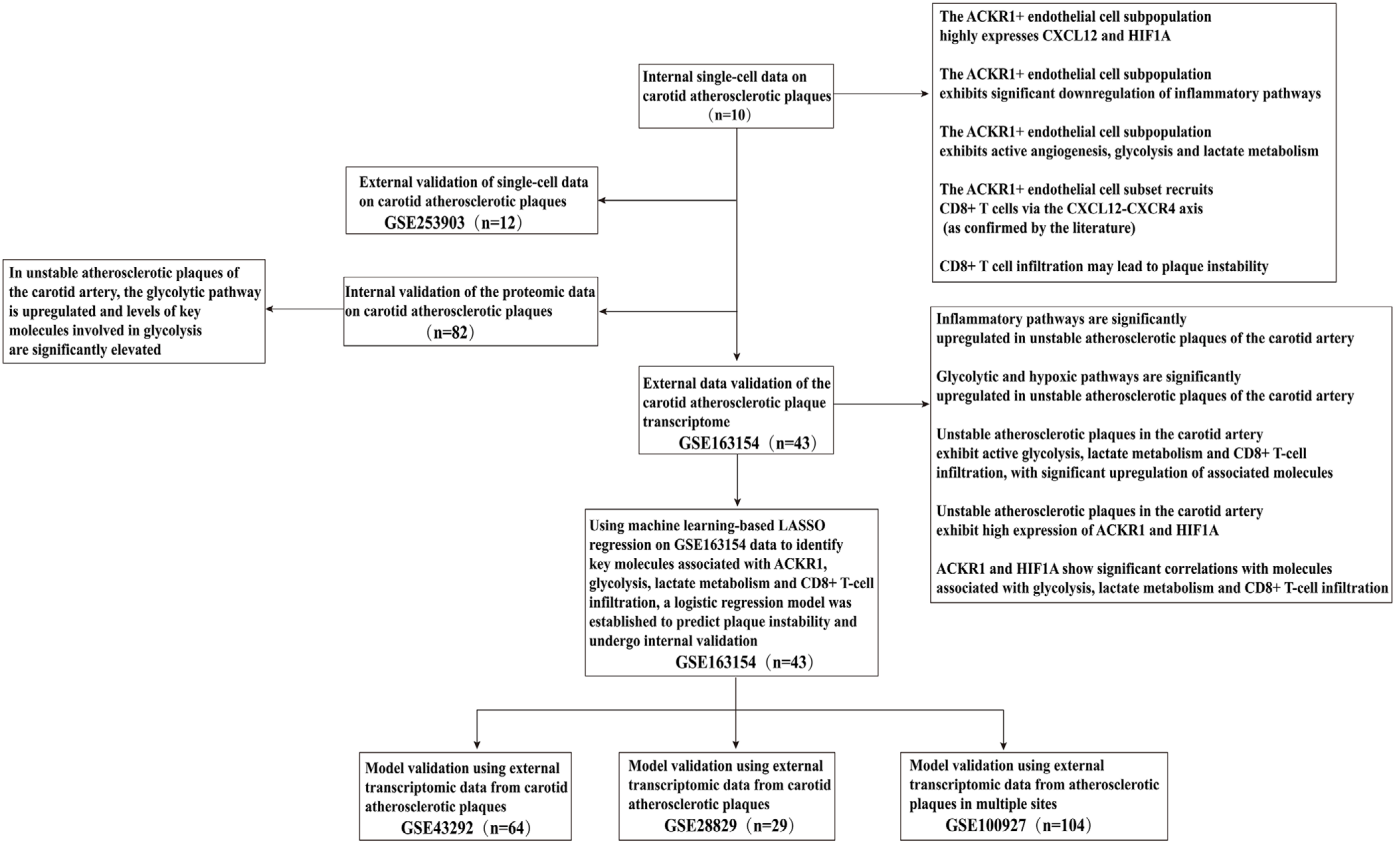
Research process flowchart.

### 2.9 Immunohistochemical staining and quantification of human carotid atherosclerotic plaques

For immunohistochemistry (IHC), CEA specimens were fixed in 4% paraformaldehyde for 24 h immediately after excision, dehydrated through a graded ethanol series, cleared in xylene and embedded in paraffin. Serial 4‐µm sections were mounted on adhesion slides and baked at 60°C for 1 h. For staining, sections were deparaffinised in xylene, rehydrated through graded ethanol to distilled water, and subjected to microwave antigen retrieval in EDTA antigen retrieval buffer (pH 9.0) for 15 min. After natural cooling to room temperature, sections were washed three times with PBS (pH 7.4) for 5 min each, incubated with 3% H_2_O_2_ at room temperature in the dark for 25 min to quench endogenous peroxidase activity, washed and blocked with 3% bovine serum albumin at room temperature for 30 min.

Sections were incubated overnight at 4°C in a humidified chamber with the following primary antibodies: anti‐ACKR1 (Proteintech, 55185‐1‐AP), anti‐CD8 (Servicebio, GB12068‐50), anti‐HIF‐1α (Abcam, ab8366), anti‐SLC16A3/monocarboxylate transporter 4 (MCT4; Abcam, ab308528), anti‐SLC16A4/MCT5 (Proteintech, 20889‐1‐AP) and anti‐pan‐lysine lactylation (Pan‐Kla; PTM Bio, PTM‐1401RM), all at a dilution of 1:200. On the following day, sections were rewarmed for 45 min, washed with PBS, and incubated with the corresponding horseradish peroxidase‐conjugated secondary antibodies (Servicebio, GB23204 and GB23303) at room temperature for 50 min. Colour was developed using a 3,3′‐diaminobenzidine chromogenic kit (Servicebio, G1212‐200T) under microscopic monitoring, and nuclei were counterstained with haematoxylin. Sections were then dehydrated through graded ethanol, cleared in xylene and mounted with neutral resin. PBS was used in place of the primary antibody as a negative control.

Whole slides were scanned using a digital pathology slide scanner (3DHISTECH) and viewed with SlideViewer, and quantification was performed using Saiviewer (version 3.0.1) and Image‐Pro Plus 6.0. IHC quantitative data were analysed with each plaque as an independent statistical unit (stable plaque group, *n* = 5; unstable plaque group, *n* = 5). Quantification strategies varied according to the spatial distribution of each marker: ACKR1 and CD8 were quantified separately in three regions of interest (ROIs) selected within each plaque to capture intraplaque spatial heterogeneity; HIF‐1α, SLC16A3 and SLC16A4 were quantified globally across the entire intimal region, with three independent repeated measurements of the same region to control for measurement fluctuations of automated image analysis; and Pan‐Kla was quantified separately in two anatomical compartments, the lipid core and the fibrous cap, with a single measurement per case. ACKR1, HIF‐1α, SLC16A3 and SLC16A4 were expressed as H‐scores, CD8 as positive cell density (cells/mm^2^) and Pan‐Kla as mean optical density (IOD/Area, where IOD denotes integrated optical density). The two types of repeated measurements differ in nature and were evaluated separately. For ROI sampling, the median within‐case coefficients of variation were 38.9% for ACKR1 (range, 17.4%–63.9%) and 47.3% for CD8 (range, 13.9%–76.5%), and within‐replicate variance accounted for 18.7% and 33.4% of the total variance, respectively, indicating true intraplaque spatial heterogeneity. For global intimal quantification, the median within‐case coefficients of variation of repeated measurements were 1.7%–1.8% (range,  .7%–7.7%), and within‐replicate variance accounted for <.3% of the total variance, indicating good measurement reproducibility and confirming that the three readings were technical replicates. In both cases, the arithmetic mean of the three readings was used as the representative value of each case for between‐group comparisons; the former represents the overall plaque level, whereas the latter reduces measurement noise. The degrees of freedom for statistical tests were always based on the number of cases rather than the number of measurements to avoid artificial inflation of the degrees of freedom due to pseudoreplication.

### 2.10 Spatial transcriptomic analysis of human carotid atherosclerotic plaques

Spatial transcriptomic data from 10 in‐house carotid atherosclerotic plaque samples were included in this study. After upstream quality control filtering, raw data were loaded into R using the *Seurat* package. Count matrices were extracted for each sample and uniformly constructed as a Spatial assay, and spatial coordinates and cell‐type annotations obtained by label transfer from the single‐cell reference dataset (predicted.id) were matched by barcode. Quality control metrics included the number of detected genes, total unique molecular identifier counts, the proportion of mitochondrial genes, and the proportion of haemoglobin genes per spot (percent.hb; gene set: *HBA1*, *HBA2*, *HBB*, *HBD*, *HBM*, *HBQ1*, *HBG1*, *HBG2*, *HBZ* and *HBE1*); because ACKR1 is highly expressed in erythrocytes, percent.hb was used to identify blood contamination. Expression matrices were normalised using the LogNormalise method (scale factor = 10 000). Samples whose gene panels did not cover *ACKR1* were excluded from analyses involving *ACKR1*.

#### 2.10.1 Definition of cell populations

ACKR1^+^ EC spots were required to meet three criteria simultaneously: annotation as ECs by predicted.id, normalised *ACKR1* expression >0, and percent.hb <20%; the third criterion was used to exclude false‐positive signals caused by erythrocyte contamination. CD8^+^ T‐cell spots were defined on the basis of marker gene expression, requiring expression >0 of at least one of *CD3D* and *CD3E* and of at least one of *CD8A* and *CD8B*.

#### 2.10.2 Spatial proximity analysis

Analyses were performed independently within each sample. Using the coordinates of all ACKR1^+^ EC spots in a sample as the reference point set, the Euclidean distance from each CD8^+^ T‐cell spot to the nearest ACKR1^+^ EC spot was calculated using the *get.knnx* function in the *FNN* package (*k* = 1). Meanwhile, an equal number of spots was randomly sampled from all spots in the same sample (set.seed = 1) as controls, and their distances were calculated using the same method. To eliminate between‐sample differences in capture area scale and ACKR1^+^ EC density, the median distance of the random control spots was used as a normalisation factor to convert both sets of distances into relative distances (random control median = 1), which were then pooled for analysis. Samples with fewer than five ACKR1^+^ EC or CD8^+^ T‐cell spots were excluded. Between‐group comparisons were performed using the Wilcoxon rank‐sum test, and a two‐sided *p* < .05 was considered statistically significant. The results were presented as spatial scatter plots, sample‐wise cumulative distribution curves of distances, and pooled violin–box plots; plots were generated using *ggplot2*, and scatter layers were rasterised using *ggrastr*.

### 2.11 Statistical analysis

All data processing and statistical analyses were performed using R software (version 4.3.1); the availability of analysis scripts and processed data is described in the ‘Data Availability Statement’. Continuous variables were compared between groups (e.g., feature gene expression or module scores between stable and unstable plaques) using independent‐samples Student's *t*‐tests or non‐parametric Wilcoxon rank‐sum tests. Pearson correlation analysis was used to evaluate correlations between variables and to calculate the Pearson correlation coefficient (*r*). ROC curves were plotted and AUCs with 95% CIs were calculated using the *pROC* R package. A two‐sided *p* value <.05 was considered statistically significant for all tests. Multiple comparisons were controlled for the FDR using the BH method. Categorical variables were compared between groups using Fisher's exact test. Conditional LR, mixed‐effects models, and Firth penalised LR were performed using the *survival*, *lme4* and *logistf* packages, respectively.

#### 2.11.1 Statistical analysis of IHC quantitative indices

The statistical strategy was prespecified before analysis. H‐scores and positive cell densities are ratio‐scale indices that showed right‐skewed distributions, with within‐group variability increasing with the mean (between‐group variance ratios ranging from  .27 to 38.8, four of which exceeded 10‐fold), consistent with log‐normal distributions; therefore, all were log_10_‐transformed (all observations were >0, so no constant was added). After transformation, the maximum deviation of the variance ratio from 1 decreased from 38.8‐fold to 5.1‐fold, indicating a marked improvement in heteroscedasticity. Pan‐Kla optical density values (IOD/Area) were concentrated within a narrow range of 167–174 and represent an approximately interval scale; because the difference in *p* values before and after log transformation was <.001, these values were analysed on the original scale.

Normality was assessed using the Shapiro–Wilk test, and homogeneity of variance was assessed using the median‐based Levene's test (Brown–Forsythe variant). Between‐group differences were assessed using independent‐samples *t*‐tests (two‐tailed, pooled variance). Mean differences on the log scale were back‐transformed and expressed as geometric mean ratios (GMRs; unstable/stable) with 95% CIs to reflect fold changes between groups. Effect sizes were expressed as Cohen's *d*. Given that seven molecular markers were tested simultaneously, the FDR was controlled using the BH method, and adjusted *q* values were reported, with *q* < .05 considered statistically significant. All statistical analyses were performed in R, and graphs were generated using GraphPad Prism.

Of note, with only five samples per group, the Shapiro–Wilk and Levene's tests have very low statistical power; a *p* > .05 indicates only a lack of evidence against the null hypothesis and does not confirm normality or homogeneity of variance. Distributional assumptions in this study were therefore based primarily on the measurement‐scale properties of each index rather than on test results. In the stable plaque group, CD8 still deviated from normality after log transformation (Shapiro–Wilk *p* = .023); however, the raw‐scale *t*‐test, log‐scale *t*‐test and Mann–Whitney *U*‐test yielded consistent conclusions, indicating that this result was insensitive to the choice of analytical method. These IHC results are exploratory and require validation in larger samples.

## 3 RESULTS

### 3.1 ACKR1 exhibits endothelial‐specific expression within the plaque and ACKR1^+^ ECs are highly correlated with intraplaque neovascularisation

We performed a series of computational analyses on our in‐house scRNA‐seq data, as illustrated in Figure 2. First, UMAP dimensionality reduction of the 10 in‐house samples was conducted, identifying a total of 18 cell subclusters (Clusters 0–17; Figure 2A). Based on the expression patterns of canonical marker genes from established literature, these clusters were manually annotated into specific cell types (refer to Table 1). The identified cell types for each cluster (Figure 2B) were: T cells (Clusters 0, 4, 10, 15); NKT cells (1); monocytes (6); macrophages (3, 5); cDC2 (9); pDCs (17); ECs (7, 11); SMCs (2, 16); B cells (8); plasma cells (14); mast cells (13); and proliferative cells (12).

**FIGURE 2:**
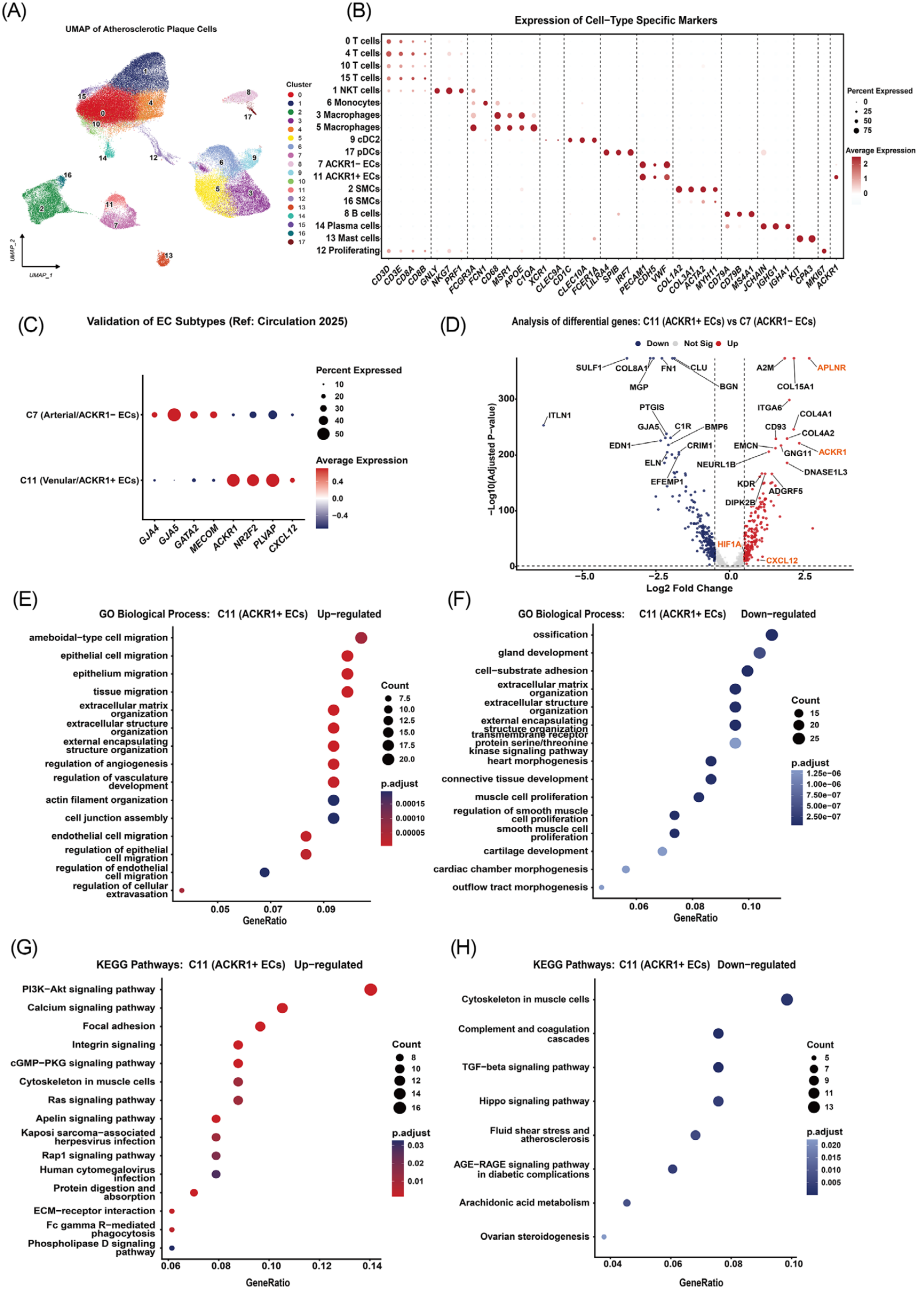
Identification and functional characterisation of the ACKR1^+^ endothelial cell subpopulation. (A) Uniform manifold approximation and projection (UMAP) plot of the cell subclusters from the in‐house scRNA‐seq data. (B) Cell‐type annotation of the identified subclusters. (C) Identification of specific endothelial cell phenotypes for Cluster 7 (ACKR1^−^ ECs) and Cluster 11 (ACKR1^+^ ECs) based on relevant literature. (D) Differential gene expression analysis of Cluster 11 (ACKR1^+^ ECs) relative to Cluster 7 (ACKR1^−^ ECs). (E) Significantly upregulated Gene Ontology (GO) pathways in Cluster 11 (ACKR1^+^ ECs) compared with Cluster 7 (ACKR1^−^ ECs). (F) Significantly downregulated GO pathways for the same comparison. (G) Significantly upregulated Kyoto Encyclopedia of Genes and Genomes (KEGG) pathways in Cluster 11 (ACKR1^+^ ECs) compared with Cluster 7 (ACKR1^−^ ECs). (H) Significantly downregulated KEGG pathways for the same comparison.

Within the endothelial population, we identified two distinct subclusters: Cluster 7 and Cluster 11. Analysis of *ACKR1* gene expression (Figure 2B) revealed that Cluster 11 exhibited prominent high expression of *ACKR1*, whereas Cluster 7 showed a significantly lower level of *ACKR1* expression. Notably, *ACKR1* was nearly absent in all other cell types within the carotid atherosclerotic plaques, indicating that its expression is highly specific to ECs. This specificity provided a rational basis for our subsequent correlation analyses using bulk RNA‐seq data. Following the research by Parma et al. regarding intraplaque neovascularisation‐mediated CD8^+^ T‐cell recruitment, Cluster 11 was annotated as ACKR1^+^ ECs, while Cluster 7 was annotated as ACKR1^−^ ECs (Figure 2C). This annotation was based on the differential expression features between the two clusters rather than on an arbitrarily defined expression threshold: *ACKR1* was significantly and specifically upregulated in Cluster 11 (avg log_2_FC = 2.37, adjusted *p* = 8.57e−222), with a proportion of positive cells of 51.1% versus 13.5% in Cluster 7 (Supporting Information Figure 5A,B). Accordingly, ‘+/−’ in this article denotes relative enrichment between the two subpopulations, and ACKR1^−^ ECs still display low‐level basal expression.

Differential expression analysis between ACKR1^−^ ECs (Cluster 7) and ACKR1^+^ ECs (Cluster 11) was further performed using the *FindMarkers* function in *Seurat* (based on the Wilcoxon rank‐sum test), together with GO and KEGG pathway enrichment analyses. *APLNR* (apelin receptor), a gene closely associated with angiogenesis and its regulation, was significantly upregulated in ACKR1^+^ ECs (Figure 2D), suggesting active neovascularisation within this subpopulation. In addition, *CXCL12* was also significantly higher in the ACKR1^+^ endothelial subpopulation than in ACKR1^−^ ECs (log_2_FC = .968, adjusted *p* = 1.2e−12; positive rate 21.2% vs. 13.1%), consistent in direction with previous reports. It should be noted that this difference exists within the endothelial compartment; across all cell types, the principal source of *CXCL12* was SMCs (Table S6). Additionally, we observed substantial expression of *HIF1A* (hypoxia‐inducible factor 1 subunit alpha), indicating that this subpopulation may be undergoing hypoxia‐induced stress.

The upregulated (Figure 2E) and downregulated (Figure 2F) GO biological processes in ACKR1^+^ ECs were identified. Significant enrichment was observed in pathways such as epithelial cell migration, regulation of angiogenesis and regulation of vasculature development, suggesting the active angiogenic profile of this subpopulation. Similarly, KEGG pathway enrichment analysis revealed downregulated pathways (Figure 2H) and an upregulation of the apelin signalling pathway (Figure 2G), further supporting the close association between ACKR1^+^ ECs and intraplaque neovascularisation. These results demonstrate a high degree of consistency with the conclusions reported by Parma et al.

In addition, because the subsequent model training cohort is defined by IPH, we examined the expression of the IPH markers *BLVRB*, *HMOX1* and *CD164* between ACKR1^+^ ECs (Cluster 11, *n* = 1806 cells) and ACKR1^−^ ECs (Cluster 7, *n* = 3885 cells) in the single‐cell data. All three showed low positive rates in both endothelial populations, and none reached significance after multiple testing correction: *BLVRB*, 7.9% versus 11.8% (avg log_2_FC = −.172, adjusted *p* = .145); *HMOX1*, 7.2% versus 6.5% (log_2_FC = .126, adjusted *p* = 1.000); and *CD164*, 18.4% versus 19.9% (log_2_FC = −.046, adjusted *p* = 1.000; Supporting Information Figure 6D–F).

We also systematically examined the distribution of the glycolysis, hypoxia and lactate metabolism module scores across all cell types in the single‐cell data. All module scores were highest in macrophages (Cluster 3) rather than in ACKR1^+^ ECs: the glycolysis score was  .500 in macrophages versus  .063 in ACKR1^+^ ECs (ranking seventh among 18 cell types), and the lactate metabolism score was negative in ACKR1^+^ ECs (−.060, ranking 13th). With respect to the hypoxia score, although ACKR1^+^ ECs ranked third, their absolute value (.009) was only about one‐thirtieth of that of macrophages (.294). This indicates that a hypoxic signal is indeed detectable in this subpopulation, consistent with the tissue microenvironment in which neovascular endothelium resides, but that its intensity is far lower than in myeloid cells (Supporting Information Figure 6H).

### 3.2 ACKR1^+^ ECs exhibit downregulated inflammatory pathways alongside hyperactive glycolysis and lactate metabolism, and are associated with the local enrichment of CD8^+^ T cells

To enhance the reliability of our findings, we further performed GSEA on ACKR1^+^ ECs based on the HALLMARK database. As shown in Figure 3A, GSEA revealed a significant downregulation of the ‘TNFA SIGNALLING VIA NFKB’ inflammatory pathway in ACKR1^+^ ECs. Within the inflammatory microenvironment of atherosclerosis, the downregulation of inflammatory pathways in an ACKR1^+^ EC subpopulation that is closely associated with neovascularisation is a noteworthy observation. To further investigate this phenomenon, we analysed TF activity within ACKR1^+^ ECs. The results indicated a significant increase in the activity of *GLI2*, a TF highly associated with glycolysis (Figure 3B). Furthermore, the HALLMARK‐based OXPHOS pathway activity score (Figure 3C) also showed a significant reduction in OXPHOS levels in this subpopulation (*p* = .0035). Comparison of core glycolytic enzymes between ACKR1^+^ ECs and ACKR1^−^ ECs (Figure 3D) revealed that the expression levels of most core glycolytic enzymes were elevated in ACKR1^+^ ECs.

**FIGURE 3:**
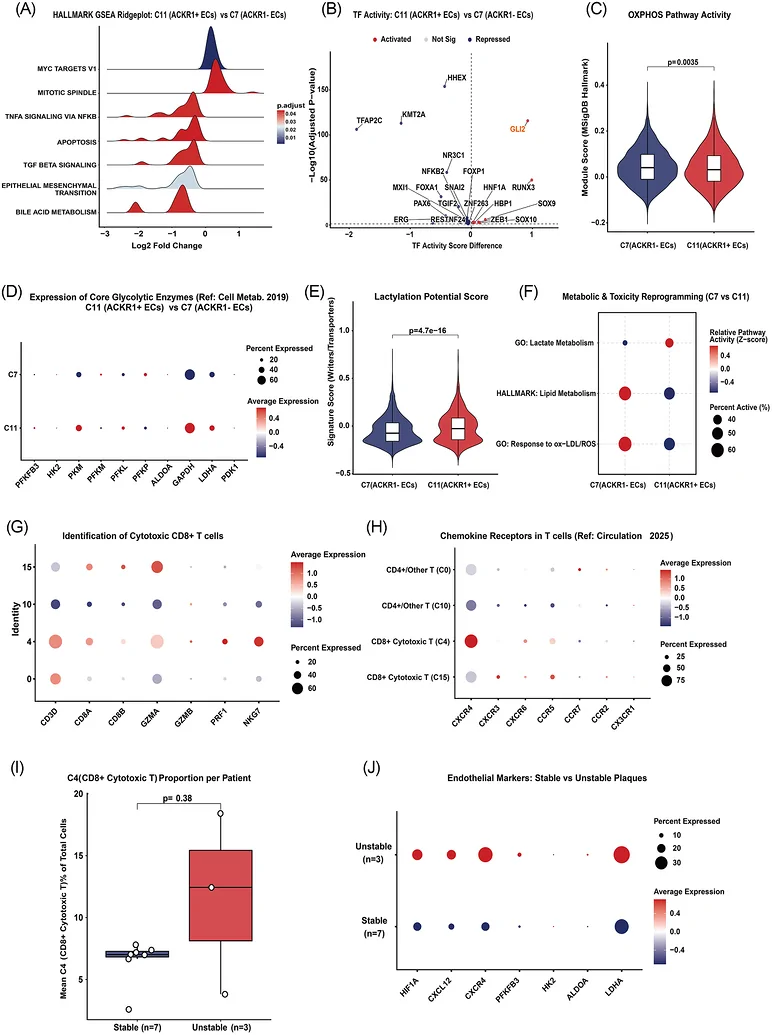
Lactate metabolism in ACKR1^+^ endothelial cells and associated CD8^+^ T‐cell infiltration. (A) Gene set enrichment analysis (GSEA) of ACKR1^+^ ECs relative to ACKR1^−^ ECs. (B) Transcription factor activity analysis. (C) Oxidative phosphorylation (OXPHOS) pathway activity analysis based on the HALLMARK database. (D) Comparison of the expression of core glycolytic enzymes. (E) Comparison of module scores based on core lactylation genes for the assessment of lactate metabolism. (F) Comparison of lactate metabolism, lipid metabolism and responsiveness to ox‐LDL/reactive oxygen species based on the GO and HALLMARK databases. (G) Identification of CD8^+^ T‐cell subclusters based on canonical marker expression. (H) Comparison of the expression of T‐cell‐related receptors across different T‐cell subclusters. (I) Comparison of the proportion of Cluster 4 (*CXCR4*‐high CD8^+^ T cells) between the stable and unstable plaque groups. (J) Comparison of the expression of key molecules between the stable and unstable plaque groups.

Considering that lactate derives principally from glycolytic flux, we performed direct module scoring of glycolytic activity using a method identical to that used for the OXPHOS analysis (Supporting Information Figure 5C,D). At the level of glycolytic rate‐limiting enzymes, the score of ACKR1^+^ ECs was higher than that of ACKR1^−^ ECs (median  .0494 vs. −.0147; Cliff's *δ* = +.199, *p* = 1.28e−33), a direction consistent with the differential expression results above, with the GSEA enrichment of HALLMARK_GLYCOLYSIS in the bulk data (Supporting Information Figure 2G), and with the upregulation of glycolytic proteins in the proteomic data (Figure 6H). It should be noted that the broad HALLMARK_GLYCOLYSIS score differed only minimally and in the opposite direction between the two subpopulations (median  .0136 in ACKR1^−^ ECs vs. −.0007 in ACKR1^+^ ECs; Cliff's *δ* = −.204, *p* = 1.71e−35); the absolute effect sizes of both comparisons were only of ‘small’ magnitude (|*δ*| ≈  .20), and their extremely low *p* values are attributable mainly to the sample size of thousands of cells. This apparently opposite direction can be explained by the compositional heterogeneity of the gene set itself: among the 198 genes actually detected in the present data object, only 17 (approximately 8.6%) are true glycolytic catalytic enzymes, with the remainder largely related to biosynthesis and regulation, so that the aggregate score mainly reflects non‐glycolytic biological processes. Gene‐by‐gene tracing showed that none of the top 15 driver genes elevating the broad score of ACKR1^−^ ECs was a glycolytic rate‐limiting enzyme; this reverse difference was instead dominated by growth‐factor‐binding and stress‐response genes such as *IGFBP3* (mean expression 10.004 vs. 1.881) and *IER3* (10.207 vs. 3.702), which are intrinsically highly expressed in arterial‐type ACKR1^−^ ECs (Figure 2D). Targeted scoring of rate‐limiting enzymes therefore better reflects genuine glycolytic catalytic flux, which is why the present study focuses on the core rate‐limiting enzymes.

These findings suggest active glycolytic metabolism within ACKR1^+^ ECs. Since anaerobic glycolysis in ECs is typically accompanied by elevated lactate levels, we constructed a 12‐gene lactylation signature to comprehensively assess metabolic remodelling at single‐cell resolution. This gene set systematically covers the entire biological process of lactate metabolism and epigenetic modification, including key lactate‐generating enzymes (*LDHA*, *LDHB*), microenvironmental lactate transport hubs MCT1/4 (*SLC16A1*, *SLC16A3*), core writers of lactylation (*EP300*, *KAT2A*) and erasers mediating dynamic delactylation (*HDAC1‐3*, *SIRT1‐3*). Scoring this module accurately reflects the integrated levels of intracellular lactate homeostasis and lactylation‐related epigenetic activity. The score for this module (Figure 3E) was significantly higher in ACKR1^+^ ECs compared with ACKR1^−^ ECs (*p* = 4.7e−16). Furthermore, the GO‐based lactate metabolism pathway score (Figure 3F) was also significantly higher in ACKR1^+^ ECs, indicating active lactate metabolic pathways in this subpopulation.

We further examined the expression distribution of the two key monocarboxylate transporters across the cell types of the plaque. The cell types with the highest *SLC16A4* expression were SMCs (mean expression  .172, positive rate 17.4%) and ACKR1^−^ ECs (.138, 10.6%), whereas *SLC16A3* was mainly enriched in macrophages (mean expression  .840, positive rate 62.1%) and other myeloid cells, indicating markedly different cellular origins for the two (Table S5). Even when compared only within the endothelial compartment, *SLC16A4* was higher in ACKR1^−^ ECs than in ACKR1^+^ ECs (10.6% vs. 4.4%). Given that SMCs are a core component of the fibrous cap of stable plaques, their loss during lesion progression may to a considerable extent account for the decline in overall tissue *SLC16A4* expression.

Referring to the study by Parma et al. regarding the recruitment of CD8^+^ T cells via the CXCL12–CXCR4 axis by intraplaque ACKR1^+^ ECs, we validated this mechanism in our scRNA‐seq data. Our analysis identified four T‐cell subclusters (0, 4, 10 and 15). Based on marker annotation (Figure 3G), we confirmed Clusters 4 and 15 as CD8^+^ T cells and observed that CD8^+^ T cells in Cluster 4 highly expressed *CXCR4* (Figure 3H), consistent with the report of the aforementioned study.

However, the study by Parma et al. only identified the chemotactic recruitment potential of this subpopulation at the single‐cell level and did not further confirm whether this process is associated with plaque instability. In our study, we compared the proportion of Cluster 4 CD8^+^ T cells between stable and unstable patient groups based on single‐cell data. At the single‐cell patient‐group level, the proportion of CXCR4‐high CD8^+^ T cells (Cluster 4) showed an increasing trend in the unstable group, but the between‐group difference did not reach statistical significance (*p* = .38, Figure 3I); given that the unstable group comprised only three cases, this result is statistically underpowered and is reported solely as an exploratory trend rather than as a basis for causal inference. Additionally, we compared the expression of *HIF1A*, *CXCL12* and core glycolytic enzymes at the patient level. The results showed that the expression levels of these molecules were higher in unstable patients than in stable patients (Figure 3J). These single‐cell observations suggest a possible association between the two, an association supported principally by the subsequent larger bulk transcriptomic cohort, protein quantification and spatial evidence.

### 3.3 Independent validation of findings in the external scRNA‐seq dataset GSE253903

To strengthen the reliability of our conclusions, we retrieved the external scRNA‐seq dataset GSE253903 from the GEO database, which contains profiles of carotid atherosclerotic plaque samples from six symptomatic and six asymptomatic individuals, and used it for a series of validations. While the clinical grouping of this dataset (symptomatic vs. asymptomatic) does not perfectly overlap with our internal grouping (stable vs. unstable), such that some patient‐level results may differ, the inherent consistency of the plaque microenvironment makes it an entirely reasonable cohort for external validation at the single‐cell level.

UMAP dimensionality reduction was performed on this dataset (Supporting Information Figure 1A), identifying 17 distinct clusters (Clusters 0–16). Based on the expression patterns of canonical marker genes, these clusters were manually annotated into cell types (refer to Table 1). The identified cell types (Supporting Information Figure 1B) were: T cells (Cluster 0); CD8^+^ T cells (2); monocytes (1, 6); macrophages (5); cDC2 (8); pDCs (16); ECs (7, 10); SMCs (3, 4, 13, 14); B cells (15); plasma cells (11); mast cells (12); and proliferative cells (9).

Consistent with our internal data, two EC subclusters were identified in this dataset: Cluster 7 (ACKR1^+^ ECs) and Cluster 10 (ACKR1^−^ ECs). Because the external dataset underwent independent unsupervised clustering, its subpopulation numbering does not correspond to that of the internal dataset. In the internal data, ACKR1^+^ ECs are Cluster 11, whereas in GSE253903 they are Cluster 7; in the internal data, ACKR1^−^ ECs are Cluster 7, whereas in GSE253903 they are Cluster 10.

Differential expression analysis of ACKR1^+^ ECs relative to ACKR1^−^ ECs supported our previous findings, with both *APLNR* and *CXCL12* significantly elevated in ACKR1^+^ ECs (Supporting Information Figure 1C). Referring likewise to the study by Parma et al. regarding intraplaque neovascularisation‐mediated CD8^+^ T‐cell recruitment, Cluster 7 was identified as ACKR1^+^ ECs, while Cluster 10 was annotated as ACKR1^−^ arterial ECs (ACKR1^−^ aECs; Supporting Information Figure 1D).

Further GO and KEGG analyses were likewise consistent with our results described above. Supporting Information Figure 1E,G shows that ACKR1^+^ ECs exhibit active EC migration and neovascularisation (sprouting angiogenesis and the apelin signalling pathway), whereas Supporting Information Figure 1F,H indicates significant downregulation in this subpopulation of the fluid shear stress and atherosclerosis pathway as well as inflammatory signalling (the TNF and NF‐kappa B signalling pathways). In Supporting Information Figure 1I, we specifically compared key genes between the two subclusters, including the core genes (*ACKR1*, *CXCL12*), an angiogenesis marker (*VEGFR2*), a glycolytic core enzyme related to lactate production (*ENO1*), and lactate transporters (*SLC16A7*/MCT2, *SLC2A3*/GLUT3); all showed an increasing trend, indicating active angiogenesis, glycolysis and lactate metabolism in this subpopulation. Furthermore, we compared the expression of *HIF1A* and its associated prolyl hydroxylase *EGLN1* (*PHD2*). The *HIF1A* transcript showed an increasing trend in ACKR1^+^ ECs (avg log_2_FC = .265, unadjusted *p* = 8.74e−06), but did not reach significance under the genome‐wide Bonferroni correction applied by default in *Seurat* (adjusted *p* = .32), and is therefore reported only as a trend. In contrast, *EGLN1* (*PHD2*) was significantly decreased in ACKR1^+^ ECs (avg log_2_FC = −.221, adjusted *p* = 4.95e−07), whereas *EGLN2*, *EGLN3*, *VHL* and *HIF1AN* showed no significant differences. EGLN1 catalyses the prolyl hydroxylation of HIF‐1α, a modification that leads to its recognition by the E3 ubiquitin ligase VHL and subsequent proteasomal degradation; its reduced expression suggests that the degradation pathway of HIF‐1α may be attenuated in this subpopulation. It should be noted that these remain observations at the transcriptional level; evidence at the HIF‐1α protein level is presented in Section 3.8 and Figure 9G. Finally, comparisons of CD8^+^ T‐cell markers and receptors (e.g., *CXCR4*) yielded results consistent with our internal observations (Supporting Information Figure 1J).

In addition, we compared the proportion of cytotoxic CD8^+^ T cells between symptomatic and asymptomatic patients in this external cohort (six cases each). No significant difference was observed between the two groups (symptomatic group: mean 11.45%, median 6.32%; asymptomatic group: mean 11.40%, median 11.99%; Wilcoxon test *p* = .485), and the dispersion of individual values in the symptomatic group was considerable (1.7%–26.8%; Supporting Information Figure 6C). In the internal single‐cell cohort, this proportion likewise showed only a non‐significant increasing trend (*p* = .38). We consider that this result mainly reflects the limited power of single‐cell proportion estimation under small sample sizes and differences in sampling site; the conclusions of this study regarding CD8^+^ T cells rest not on cell proportions but on the expression of cytotoxic effector molecules at the bulk level (see subsequent sections).

### 3.4 The external transcriptomic cohort GSE163154 suggests upregulation of ACKR1, HIF1A, glycolysis, lactate metabolism and CD8^+^ T‐cell infiltration‐related molecules in IPH plaques, alongside significantly activated inflammatory, glycolytic and hypoxic pathways

To further expand and validate our findings, we retrieved the GSE163154 cohort from the GEO database, which comprises 43 IPH and non‐IPH carotid plaque samples from patients who underwent CEA. Because this clinical classification corresponds to the stable/unstable grouping in our internal scRNA‐seq cohort, validation utilising this dataset holds considerable value.

We performed an analysis of DEGs between the IPH and non‐IPH groups, with a specific focus on the key molecules identified in our single‐cell analysis. Significant up‐ and downregulation of relevant genes in the IPH group was observed, as visualised by the volcano plot (Figure 4A) and heatmap (Figure 4B). The vertical axis of the volcano plot represents −log10 (raw *p* value); the FDR‐adjusted significance of each gene is detailed in Table S7. These included our core molecules of interest (*ACKR1* and *HIF1A*), key lactate metabolism‐related genes (*LDHB*, *SLC16A3*, *SLC16A4*, *SLC16A7*), core glycolytic enzymes (*ALDOA*, *ALDOC*, *GAPDH*, *HK2*, *PFKL*, *PFKM*, *PKM*) and markers of CD8^+^ T‐cell infiltration (*CD8A*, *GZMA*, *GZMB*, *IFNG*, *PRF1*, *CXCR4*). Figure 4C–G presents quantitative comparisons of the expression of the above genes, intuitively demonstrating the magnitude of the differences.

**FIGURE 4:**
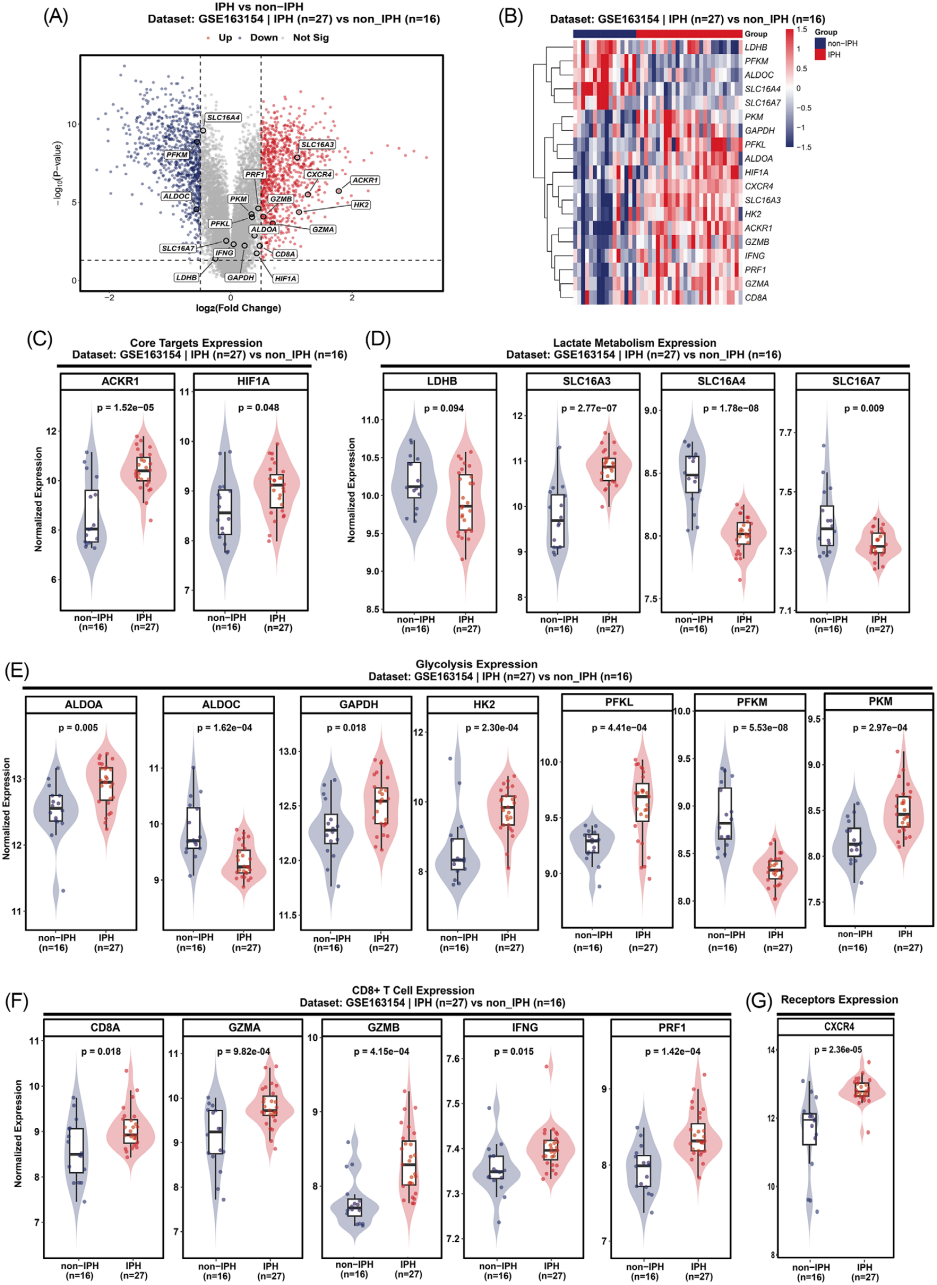
Validation of atypical chemokine receptor 1 (ACKR1), lactate metabolism and immune signatures in intraplaque haemorrhage (IPH). (A) Volcano plot illustrating the significant up‐ and downregulation of *ACKR1*, *HIF1A*, and molecules associated with glycolysis, lactate metabolism and CD8^+^ T‐cell infiltration in the IPH group. (B) Heatmap visualising the expression profiles of these molecules. (C) Quantitative comparison of the expression levels of core target genes between the IPH and non‐IPH groups. (D) Quantitative comparison of the expression levels of lactate metabolism genes for the same groups. (E) Quantitative comparison for glycolysis‐related genes. (F) Quantitative comparison for CD8^+^ T‐cell infiltration‐related genes. (G) Quantitative comparison for *CXCR4* receptor‐related genes. Values shown in the figure are Benjamini–Hochberg‐adjusted false discovery rate (FDR) values (adj. *p*) from the *limma* differential expression analysis.

To further investigate the intraplaque microenvironment, we conducted pathway enrichment analysis of the DEGs. The GO pathways in Supporting Information Figure 2A,B revealed a pronounced inflammatory microenvironment within the plaques, characterised by highly active leukocyte migration and adhesion. Concurrently, KEGG analysis (Supporting Information Figure 2C,D) indicated the activation of lipid and atherosclerosis‐related pathways, and HALLMARK pathway analysis (Supporting Information Figure 2E,F) more intuitively demonstrated the activation of inflammatory cascades, particularly the ‘TNFA SIGNALLING VIA NFKB’ pathway, confirming an overall upregulation of inflammation within unstable plaques. However, our earlier observation that ACKR1^+^ ECs paradoxically exhibit significant downregulation of the ‘TNFA SIGNALLING VIA NFKB’ pathway while displaying relatively high expression of the chemokine *CXCL12* further implies the existence of a highly specialised inflammatory regulatory system within this distinct subpopulation.

We further evaluated the status of glycolysis‐ and hypoxia‐related pathways using GSEA. The results (Supporting Information Figure 2G,H) revealed that both the ‘GLYCOLYSIS’ and ‘HYPOXIA’ pathways were significantly upregulated. This supports our scRNA‐seq findings, demonstrating the presence of a highly glycolytic and hypoxic intraplaque environment, which establishes the necessary conditions for lactate accumulation and robust *HIF1A* expression.

### 3.5 The external transcriptomic cohort GSE163154 and in‐house proteomics suggest significant correlations of *ACKR1* and *HIF1A* with glycolysis, lactate metabolism and CD8^+^ T‐cell infiltration

The aforementioned analyses revealed that *ACKR1* exhibits relative endothelial specificity, being highly expressed exclusively in a specific EC subpopulation, accompanied by elevated *HIF1A* transcript levels and associated with high *CXCL12* expression and CD8^+^ T‐cell infiltration. Building upon these findings, we performed correlation analyses in the external transcriptomic cohort GSE163154 to investigate whether *ACKR1* and *HIF1A* correlate with the molecules of these pathways in a larger sample size.

In Figure 5A, a highly significant positive correlation was observed between *ACKR1* and *HIF1A* (*r* = .47, *p* = .0014), as well as between *HIF1A* and *CXCL12* (*r* = .42, *p* = .0056), suggesting that the expression level of *CXCL12* in plaque tissue is associated with that of *HIF1A*. In Figure 5B–D, we further analysed the correlations between *ACKR1* and core glycolytic enzymes; the results demonstrated significant positive correlations between *ACKR1* and *HK2*, *PKM*, *PFKL*, *ALDOA* and *GAPDH*, implying that *ACKR1* expression is accompanied by active glycolysis. In addition, *ACKR1* was significantly positively correlated with the efflux‐type lactate transporter *SLC16A3* and significantly negatively correlated with *SLC16A4* and *SLC16A7* (Figure 5E), suggesting that the expression level of *ACKR1* in plaque tissue is accompanied by an imbalance in the expression of the lactate transporter network. It should be noted that this set of associations was markedly attenuated after adjustment for cell composition (Table S4), and therefore mainly reflects covariation at the level of cell abundance; evidence for intracellular lactate accumulation within ACKR1^+^ ECs derives principally from the single‐cell glycolysis and lactylation module scores (Figure 3D,E) and from in situ Pan‐Kla staining (Figure 10G–J). Figure 5F further verified significant positive correlations between *ACKR1* and core molecules of CD8^+^ T‐cell infiltration (*CD8A*, *GZMA*, *GZMB*, *PRF1*). Figure 6A–C demonstrates significant positive correlations of *ACKR1* with the CD8_Tox_Score and with *CXCR4*, and show that the score was significantly elevated in the IPH group, corroborating the immune infiltration of CD8^+^ T cells. Figure 6D–G shows that *HIF1A* was also significantly associated with glycolysis (*HK2*, *PFKL*, *ALDOA*), lactate metabolism (*SLC16A3*) and the CD8_Tox_Score, further suggesting the synchrony of changes in *ACKR1* and *HIF1A*.

**FIGURE 5:**
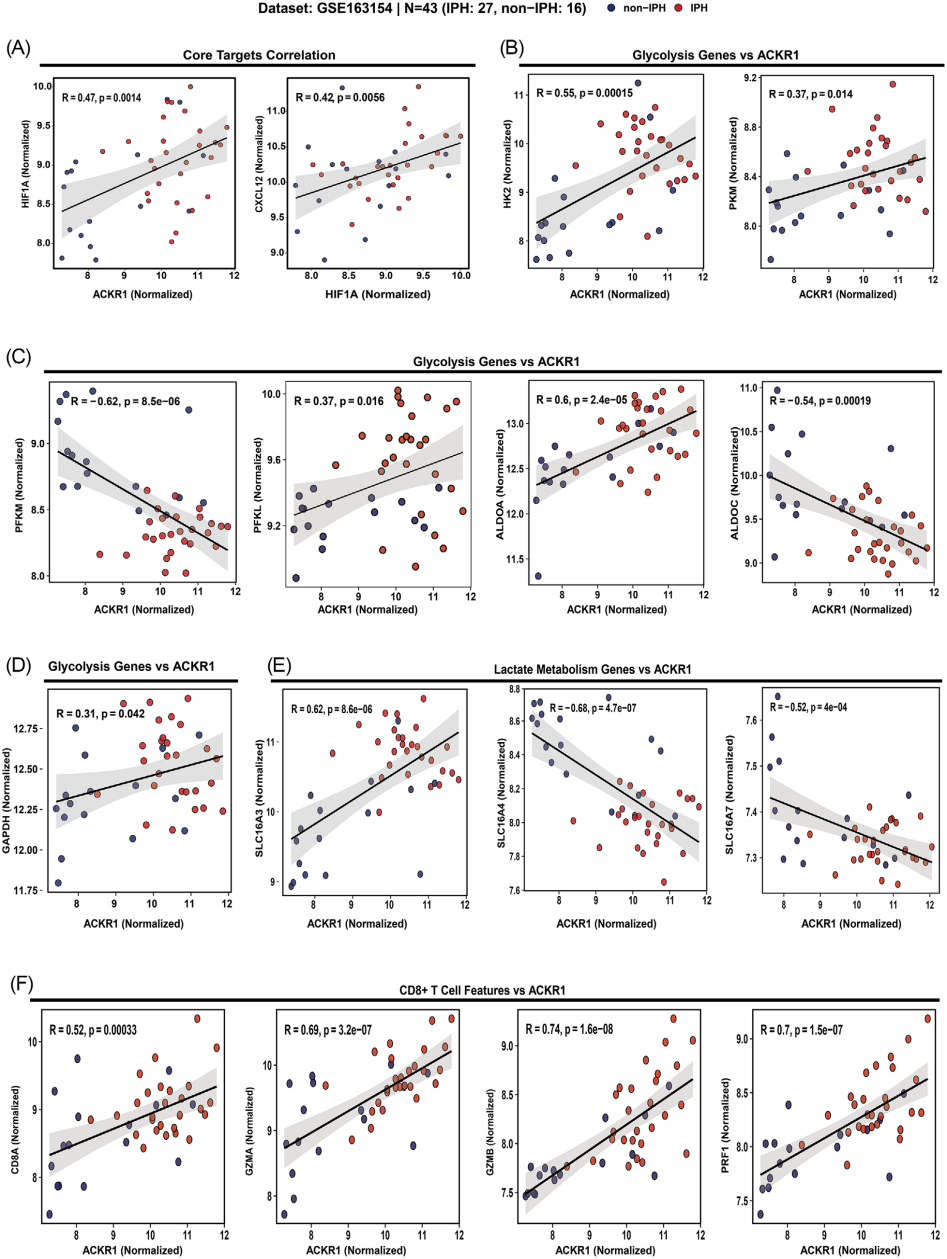
Correlation analysis based on the GSE163154 cohort. (A) Correlation of *HIF1A* with *ACKR1* and *CXCL12*. (B) Correlation between *ACKR1* and the core glycolytic enzymes *HK2* and *PKM*. (C) Correlation between *ACKR1* and the core glycolytic enzymes *PFKM*, *PFKL*, *ALDOA* and *ALDOC*. (D) Correlation between *ACKR1* and the core glycolytic enzyme *GAPDH*. (E) Correlation between *ACKR1* and core lactate metabolism molecules. (F) Correlation between *ACKR1* and core molecules associated with CD8^+^ T‐cell infiltration.

**FIGURE 6:**
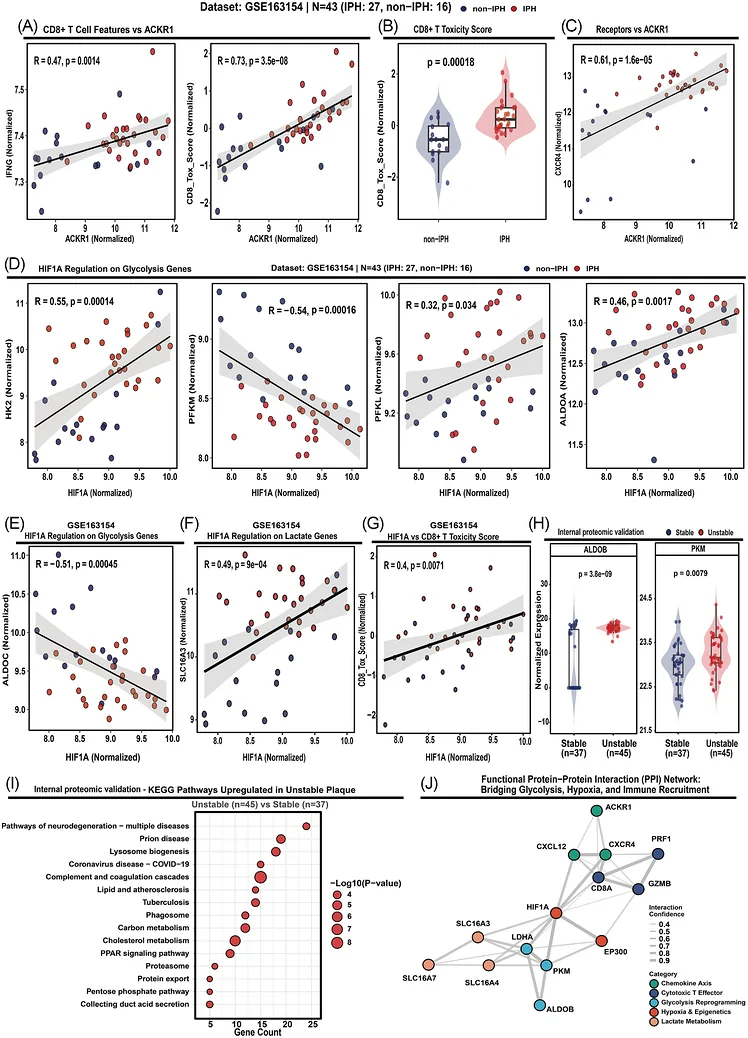
Analysis based on the GSE163154 cohort and in‐house proteomic data. (A) Correlation between *ACKR1* and CD8^+^ T‐cell signatures. (B) Comparison of the CD8^+^ T‐cell cytotoxicity score (CD8_Tox_Score) between the intraplaque haemorrhage (IPH) and non‐IPH groups. (C) Correlation analysis between *ACKR1* and *CXCR4*. (D) Correlation of *HIF1A* with *HK2*, *PFKM*, *PFKL* and *ALDOA*. (E) Correlation of *HIF1A* with *ALDOC*. (F) Correlation of *HIF1A* with *SLC16A3*. (G) Correlation of *HIF1A* with the CD8_Tox_Score. (H) Significant upregulation of the core glycolytic proteins ALDOB and PKM in unstable plaques compared with stable plaques based on in‐house proteomic data. Values shown in the figure are unadjusted *p* values; after Benjamini–Hochberg correction, ALDOB remained significant (false discovery rate [FDR] = 2.55e−11), whereas PKM did not reach the adjusted significance threshold (FDR = .053) and is therefore reported in the main text as a concordant trend. (I) Kyoto Encyclopedia of Genes and Genomes (KEGG) enrichment analysis indicating upregulated pentose phosphate and lipid and atherosclerosis pathways. (J) Protein–protein interaction (PPI) network analysis of the relevant core proteins based on the STRING database.

To assess whether the gene set significantly upregulated in ACKR1^+^ ECs (Cluster 11) relative to ACKR1^−^ ECs (Cluster 7) also differed in GSE163154, we constructed a score based on the 193 genes detectable in the bulk expression matrix. The module score was significantly higher in the IPH group than in the non‐IPH group (*p* = 1.69e−03), indicating that the transcriptional signature of this cell subpopulation is upregulated overall in IPH (Supporting Information Figure 6G).

To examine whether the above bulk correlations were affected by cell‐composition confounding, we further performed cell‐type deconvolution and partial correlation analysis (Supporting Information Figure 6B). The results showed that the correlations of *ACKR1* with some glycolytic and lactate transport molecules (*HK2*, *SLC16A3*, *SLC16A4*) were substantially attenuated and no longer significant after adjustment for cell proportions, whereas the HIF‐1α–CXCL12 relationship at the core of the mechanistic chain remained essentially unchanged before and after adjustment (partial correlation *r* = .55, *p* = .0003), suggesting that the latter reflects regulatory coupling at the intracellular level. Moreover, in the pseudo‐bulk analysis restricted to ACKR1^+^ ECs, the association between *ACKR1* and *SLC16A4* remained observable (*r* = .51, *p* = .021).

We subsequently conducted a series of validations using our in‐house proteomic data, which encompass plaque information from 37 stable and 45 unstable patients. Given the inevitable missing values inherent to proteomic detection, these results serve as a partial reference. Differential protein analysis (Figure 6H) showed that the core glycolytic protein ALDOB was significantly elevated in the unstable group (logFC = 10.83, FDR = 2.55e−11) and that PGD, a key enzyme of the pentose phosphate pathway, was also significantly elevated (logFC = .573, *p* = .0017); the glycolytic rate‐limiting enzyme PKM showed a concordant increasing trend (logFC = .269, *p* = .010, FDR = .053) that did not reach significance after multiple testing correction. It should be noted that the large fold change of ALDOB partly arises from a floor effect, as its levels were generally below the limit of detection in the stable group and were subsequently imputed; inferences here are therefore restricted to direction and significance. KEGG pathway enrichment (Figure 6I) revealed significant upregulation of the pentose phosphate pathway and the lipid and atherosclerosis pathway, confirming at the protein expression level the active glycolysis and lipid metabolism present in the unstable group.

Finally, at the protein level, to further explore the functional interplay among these proteins based on our current findings, we constructed a protein–protein interaction (PPI) network using the STRING database and the core proteins validated in this study. As shown in Figure 6J, there are tight connections among five main hubs: Hypoxia & Epigenetics, Glycolysis Reprogramming, Lactate Metabolism, Chemokine Axis and Cytotoxic T Effector. ACKR1‐associated glycolysis and lactate metabolism can link to the downstream chemotactic and immune axis via HIF‐1α.

Because this internal cohort possesses both a histopathological gold‐standard grouping and complete patient‐level clinical annotation, we further examined whether the above proteomic differences might be caused by clinical confounding. Among the 31 patient‐level variables compared, 29 showed no statistical difference between the stable and unstable groups (Table S1); the only imbalanced variable was the degree of ipsilateral carotid stenosis (severe or above: 97.8% in the unstable group vs. 75.7% in the stable group, *p* = .004), which was therefore included as a key covariate in the adjustment. After incorporating 14 clinical covariates into the design matrix, the effect sizes across the whole proteome were highly consistent with the unadjusted results (Pearson *r* of logFC = .994), and the number of proteins with *p* < .05 changed from 1193 to 1011, of which 901 (75.5%) were significant under both settings (Supporting Information Figure 7G). With respect to the specific conclusions reported in Figure 6H,I, the logFC of ALDOB changed from 10.83 to 10.50 (FDR from 2.55e−11 to 4.72e−08) and that of PGD from  .573 to  .527 (*p* from  .0017 to  .012); in the KEGG enrichment rerun on the adjusted differential proteins, the pentose phosphate pathway (*p* from 7.00e−04 to 2.25e−03) and the lipid and atherosclerosis pathway (*p* from 5.51e−04 to 1.90e−03) remained significant, with 13 upregulated pathways significant both before and after adjustment (Supporting Information Figure 7D,E and Table S3).

### 3.6 Machine learning‐based selection of core molecules related to ACKR1, glycolysis, lactate metabolism and CD8^+^ T‐cell infiltration in GSE163154 and construction of a logistic regression model for predicting plaque instability with internal validation

Having established the correlations among *ACKR1*, *HIF1A*, glycolysis, lactate metabolism and chemotactic recruitment factors for CD8^+^ T cells, as well as the link between CD8^+^ T‐cell infiltration and plaque instability, we hypothesised that a ‘glycolysis–lactate–immune’ signature derived from ACKR1^+^ ECs could serve as a predictor for carotid plaque vulnerability. To test this hypothesis, we integrated core variables identified in our previous analyses with the LASSO penalised regression algorithm to select core variables and enhance the reliability of the model.

Using the GSE163154 cohort as the training set, LASSO regression was applied to eliminate collinearity among high‐dimensional variables and perform dimensionality reduction. As shown in Figure 7A,B, the optimal penalty coefficient (*lambda.min*) was determined through 10‐fold cross‐validation; at Log *λ* = −2.922, we identified four core feature variables with the highest predictive value: *SLC16A3*, *ACKR1*, CD8_Tox_Score and *SLC16A4*. Notably, in addition to our endothelial marker *ACKR1*, molecules related to lactate metabolism and immunity were also incorporated into the signature. In the variable importance ranking of the model (Figure 7C), the lactate‐related molecules *SLC16A3* and *SLC16A4* ranked foremost, followed by *ACKR1* and the CD8_Tox_Score; furthermore, *SLC16A4* was identified as a protective factor, while the remaining three variables were risk factors. Across all 43 folds of the fully nested LOOCV, the four finally locked features were independently reselected at very high frequency: *ACKR1*, *SLC16A3* and *SLC16A4* were selected in 100% of folds and the CD8_Tox_Score in 86% of folds, indicating that this feature combination is not an incidental product of the small sample size (Supporting Information Figure 6A).

**FIGURE 7:**
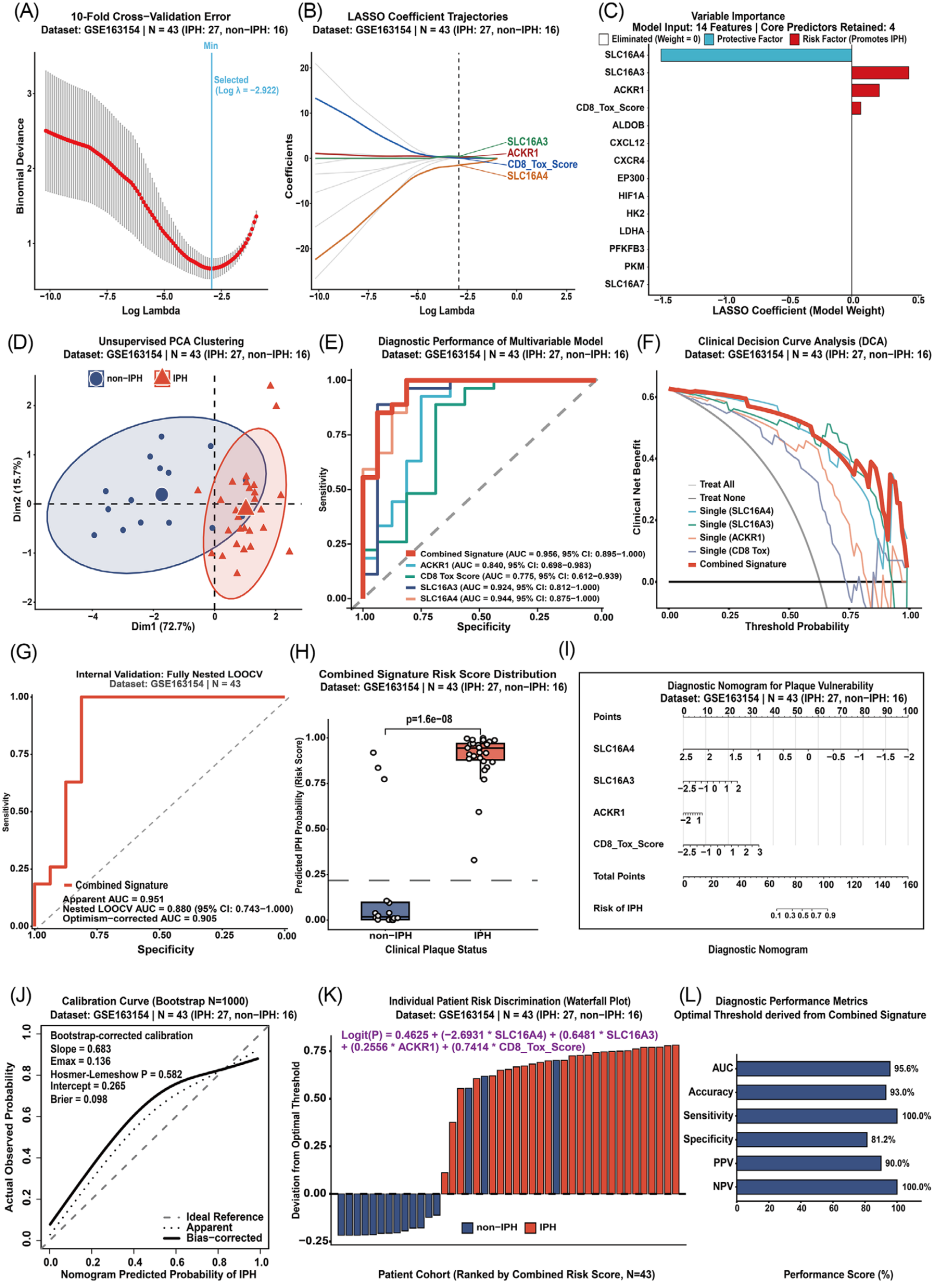
Construction and internal validation of the intraplaque haemorrhage (IPH) predictive model. (A, B) Determination of the optimal penalty parameter (*λ*) and the selected variables using least absolute shrinkage and selection operator (LASSO) regression with 10‐fold cross‐validation. (C) Variable importance ranking of the model. (D) Unsupervised principal component analysis (PCA) based on the combined model. (E) Receiver operating characteristic (ROC) curves and area under the receiver operating characteristic curves (AUC) of the individual markers and the combined model. (F) Decision curve analysis (DCA) of the individual markers and the combined model. (G) Assessment of the internal predictive performance of the combined model using leave‐one‐out cross‐validation (LOOCV). (H) Risk scores of the IPH and non‐IPH groups calculated from the combined model. (I) Nomogram illustrating the relative weight of each variable in the model; it is intended to depict the model structure and is not intended for use as a clinical risk calculator. (J) Calibration curve of the combined model, based on 1000 bootstrap resamples. (K) Waterfall plot and formula visualising the prediction for individual patients. (L) Summary of the performance metrics of the combined model. Of note, three AUC values are reported for the training cohort and are not interchangeable: The AUC in (E) and (L) is the apparent AUC of the unpenalised multivariable logistic regression model (.956); the apparent AUC in (G) is that of the LASSO‐penalised model (.951), the slight difference reflecting the different modelling approaches; and the leakage‐free estimates obtained by fully nested LOOCV and bootstrap optimism correction are  .880 and  .905, respectively (see Section 3.6).

All four selected variables showed significant between‐group differences in the training cohort: *ACKR1* (Cliff's *δ* = .681, *p* = 1.09e−04), *SLC16A3* (*δ* = .847, *p* = 3.81e−07), *SLC16A4* (*δ* = −.889, *p* = 5.44e−08) and CD8_Tox_Score (*δ* = .551, *p* = 2.25e−03; Supporting Information Figure 8A). Further dissection of the composition of the CD8_Tox_Score showed that its between‐group difference derived mainly from cytotoxic effector molecules rather than lineage markers: the between‐group differences for *GZMB*, *PRF1* and *GZMA* were *p* = 1.40e−05, 2.89e−05 and 2.47e−03, respectively, whereas the lineage marker *CD8B* showed no significant difference between groups (*p* = .390) and *CD8A* showed only a marginal difference (*p* = .040; Supporting Information Figure 8B). This suggests that what is elevated in IPH plaques is principally the cytotoxic activation state of CD8^+^ T cells rather than their number—a distinction of considerable importance for interpreting the negative results of the single‐cell proportion analysis (see Sections 3.3 and 4).

Based on these four variables, we constructed a combined signature using a LR model. To evaluate its clinical discrimination, predictive capacity, net benefit and internal overfitting, several analyses were performed. Unsupervised PCA (Figure 7D) demonstrated that the IPH and non‐IPH groups could be significantly distinguished, indicating good discrimination of the model in the training set. Comparison of ROC curves (Figure 7E) revealed that the combined signature achieved an AUC of  .956 (95% CI,  .895–1.000), which was substantially higher than those of the individual components: *SLC16A3* (AUC = .924; 95% CI,  .812–1.000), *SLC16A4* (AUC = .944; 95% CI,  .875–1.000), *ACKR1* (AUC = .840; 95% CI,  .698–.983) and CD8_Tox_Score (AUC = .775; 95% CI,  .612–.939), indicating a high capacity of the model to predict plaque instability. DCA (Figure 7F) further indicated that the clinical net benefit of the combined signature outperformed individual variables. In Figure 7G, to mitigate potential overfitting due to sample size, we applied the rigorous LOOCV method within the training set; under fully nested LOOCV the AUC was  .880 (95% CI,  .743–1.000), the mean optimism estimated from 1000 bootstrap resamplings was only  .046, and the optimism‐corrected AUC was  .905, indicating no evident overfitting.

Furthermore, in Figure 7H we calculated risk scores for both groups to further demonstrate the model's excellent discriminative performance between the two groups. We also visualised the relative contribution of each variable in the model in the form of a nomogram (Figure 7I). In addition, to assess the calibration of the model's predictive performance, we plotted a calibration curve in Figure 7J. The calibration curve was based on 1000 bootstrap resamplings. The optimism‐corrected calibration slope was  .683 and the intercept  .265; the Brier score was  .098 (apparent value  .070; 95% CI,  .021–.137); the maximum calibration error (Emax) was  .136; and the Hosmer–Lemeshow test yielded *p* = .582, indicating good agreement between the predicted probabilities and the observed values. For calibration, we take the leave‐one‐out predicted values from the nested LOOCV (free of any information leakage) as the primary measure: the calibration slope was 1.105 (95% CI,  .693–2.570), the calibration intercept −.049 (95% CI, −.798–.728) and the Brier score  .096 (Supporting Information Figure 7F).

To visually present the model, in Figure 7K we used a waterfall plot and the following diagnostic equation to visualise individual patient predictions: *Logit(p) = .4625 + (−2.6931 × SLC16A4) + (.6481 × SLC16A3) + (.2556 × ACKR1) + (.7414 × CD8_Tox_Score)*. The coefficient of *SLC16A4* is negative, indicating that lower expression corresponds to a higher probability of vulnerability. It should be emphasised that the sign of a LASSO coefficient reflects the direction of the statistical association between that variable and plaque vulnerability, representing a data‐driven discriminant weight rather than a biological claim about its causal role or transport direction. In Figure 7L, the comprehensive performance of the combined signature was summarised, including AUC (95.6%), accuracy (93.0%), sensitivity (100.0%), specificity (81.2%), positive predictive value (PPV, 90.0%) and negative predictive value (NPV, 100.0%).

### 3.7 Independent external cohort validation: The model retains discriminative ability at both the lesion‐progression stratification and disease‐identification levels

To further validate the combined signature constructed based on *ACKR1* and the lactate–immune features in the training set GSE163154, we selected three independent datasets (GSE43292, GSE28829 and GSE100927) from GEO for external validation to comprehensively evaluate the model's performance and pan‐vascular characteristics. GSE43292 contains 32 paired tissue samples from patients undergoing CEA, with each patient contributing an atherosclerotic plaque sample from the core and shoulder (Stary stages ≥ IV) and a macroscopically intact distant tissue sample (stages I and II). GSE28829 includes 13 early‐stage (pathological intimal thickening and intimal xanthoma) and 16 advanced‐stage (thin‐ or thick‐cap fibroatheroma) carotid plaque samples. GSE100927 comprises a total of 104 atherosclerotic and control arterial tissues from the carotid, femoral and infrapopliteal arteries. Notably, since our model was constructed based on pathological grading (i.e., stable vs. unstable), and the clinical groupings in the aforementioned validation cohorts may not perfectly align with this classification, the results must be interpreted with reasonable caution. It should be noted that the three validation cohorts correspond to two different levels of task: GSE43292 (Stary stage ≥ IV vs. I–II) and GSE28829 (advanced vs. early lesions) are grouped by lesion progression/severity, which is consistent with the vulnerability‐stratification positioning of this model, whereas GSE100927 consists of normal arteries and atherosclerotic lesions and therefore represents in essence a disease‐identification task rather than vulnerability discrimination. The two categories of result are accordingly interpreted separately below, and the performance in disease identification is not equated with the precision of vulnerability prediction.

In the external validation using GSE43292, the model yielded an AUC of  .807 (95% CI,  .698–.915; Figure 8A), indicating that it retains high predictive accuracy. The model‐based risk scores could accurately distinguish between the two patient groups (*p* = 1.2e−05; Figure 8D). The specific expression of the four variables is independently displayed (Figure 8E), all of which showed significant differences between the two groups: *SLC16A4* (*p* = 8.1e−05), *SLC16A3* (*p* = .00047), *ACKR1* (*p* = 6.5e−06) and CD8_Tox_Score (*p* = 5.9e−05). These results are highly consistent with our previous findings. Additionally, the calibration curve (Figure 8B) and the DCA curve (Figure 8C) are provided.

**FIGURE 8:**
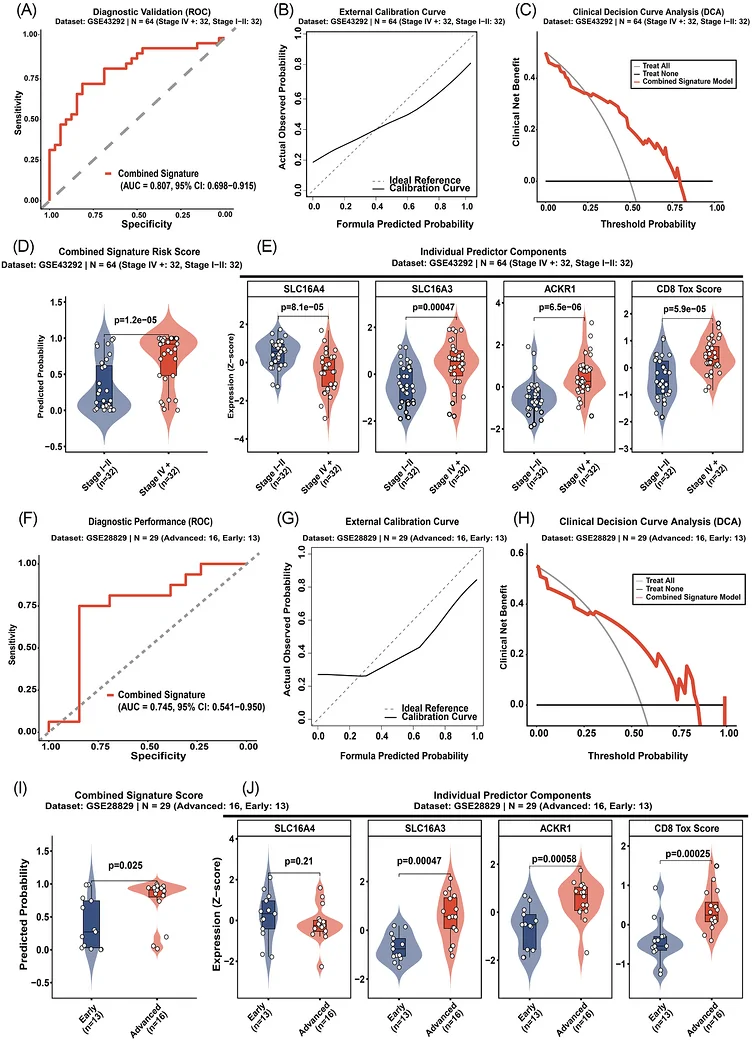
External validation of the predictive model in independent carotid atherosclerosis cohorts. (A) Receiver operating characteristic (ROC) curve for external validation in the GSE43292 cohort. (B) Calibration curve. (C) Decision curve analysis (DCA) curve. (D) Risk scores. (E) Comparison of individual variables. (F) ROC curve for external validation in the GSE28829 cohort. (G) Calibration curve. (H) DCA curve. (I) Risk scores. (J) Comparison of individual variables.

It is worth emphasising that GSE43292 employs a within‐patient paired design, in which the two paired sides derive from the same patient, so that all patient‐level variables—age, sex, symptomatic status, degree of stenosis, diabetes mellitus, lipid levels, statin and antihypertensive medication use and smoking history—are fixed by design. Under this paired structure, 28 of 32 pairs (87.5%) showed a higher risk score on the plaque side than on the intact tissue side of the same patient (exact binomial test *p* = 1.93e−05; cluster bootstrap 95% CI,  .750–.969 using the patient as the resampling unit), the paired Wilcoxon signed‐rank test yielded *p* = 2.93e−05 and the mean within‐patient increase in score was 3.91 (95% CI, 2.52–5.30). Conditional LR stratified by patient showed that each 1‐SD increase in the risk score corresponded to an OR of 7.45 (95% CI, 2.05–27.12, *p* = .0023), approximately 1.93 times the unstratified estimate (OR = 3.87; 95% CI, 1.86–8.03). That the effect was strengthened rather than attenuated after controlling for patient‐level factors suggests that between‐patient variability acts here to dilute the signal. All four model components changed within patients in the direction expected from the coefficients locked in the training set, and all were statistically significant after BH correction (Supporting Information Figure 7A–C and Table S2).

In the GSE28829 validation cohort, the model AUC was  .745 (95% CI,  .541–.950; Figure 8F). The risk scores accurately differentiated the two groups (*p* = .025; Figure 8I). The specific conditions of the four variables are illustrated (Figure 8J): *SLC16A4* (*p* = .21), *SLC16A3* (*p* = .00047), *ACKR1* (*p* = .00058) and CD8_Tox_Score (*p* = .00025). The calibration and DCA curves (Figure 8G,H) generally align with the aforementioned results.

The GSE100927 cohort encompasses the carotid, femoral and infrapopliteal arteries. First, validation in the carotid artery yielded an AUC of  .997 (95% CI,  .989–1.000; Supporting Information Figure 3A), and the risk scores accurately distinguished the two groups (*p* = 5.1e−10; Supporting Information Figure 3D). The differences in the four variables (Supporting Information Figure 3E) were: *SLC16A4* (*p* = 1.2e−06), *SLC16A3* (ns), *ACKR1* (ns) and CD8_Tox_Score (*p* = .0016). The corresponding calibration curve (Supporting Information Figure 3B) and DCA curve (Supporting Information Figure 3C) are provided. This demonstrates that the signature possesses high discrimination in distinguishing normal arteries from carotid atherosclerotic lesions. It must be emphasised that this cohort contains no pathological stratification of lesion vulnerability, so that its AUC reflects disease‐identification capacity and cannot be taken as evidence of the precision of vulnerability prediction. Second, validation in the femoral artery showed an AUC of  .846 (95% CI,  .710–.982; Supporting Information Figure 3F), with risk scores significantly distinguishing the groups (*p* = .00038; Supporting Information Figure 3I). The variable differences (Supporting Information Figure 3J) were: *SLC16A4* (*p* = .053), *SLC16A3* (ns), *ACKR1* (ns) and CD8_Tox_Score (*p* = 3.7e−07). Calibration (Supporting Information Figure 3G) and DCA (Supporting Information Figure 3H) curves are provided. Finally, validation in the infrapopliteal artery resulted in an AUC of  .779 (95% CI,  .579–.979; Supporting Information Figure 4A), with accurate group distinction via risk scores (*p* = .018; Supporting Information Figure 4D). Variable differences (Supporting Information Figure 4E) were: *SLC16A4* (*p* = .052), *SLC16A3* (ns), *ACKR1* (ns) and CD8_Tox_Score (*p* = .013). Calibration (Supporting Information Figure 4B) and DCA (Supporting Information Figure 4C) curves are presented. Collectively, these results indicate that the signature possesses high discrimination in the more basic task of ‘distinguishing the presence or absence of atherosclerotic lesions’ across all three vascular beds—carotid, femoral and infrapopliteal arteries. It must be emphasised that this cohort contains no pathological stratification of lesion vulnerability, so that its AUC reflects disease‐identification capacity and cannot be taken as evidence of the precision of vulnerability prediction.

### 3.8 Spatial transcriptomics and in situ staining support the local recruitment of CD8^+^ T cells by ACKR1^+^ ECs at the spatial and protein levels

To further validate the hypothesis that ACKR1^+^ ECs recruit CD8^+^ T cells via the CXCL12–CXCR4 axis, we first performed CellChat‐based cell–cell communication inference on the in‐house single‐cell data. The analysis successfully recovered multiple high‐abundance signals sent from ACKR1^+^ ECs to the microenvironment (such as SPP1 and MIF, Supporting Information Figure 5E,F), indicating that the analytical workflow itself is reliable; however, for the specific CXCL12–CXCR4 ligand–receptor axis, no statistically significant communication probability was obtained even in the high‐sensitivity mode with relaxed overexpressed gene thresholds, a truncated mean and a lowered minimum cell number threshold. We consider this negative result to arise more probably from the intrinsic limitations of expression‐matrix‐based probabilistic algorithms. On the one hand, single‐cell data exhibit marked sparsity and dropout effects, and chemokine receptors (particularly *CXCR4*) are generally detected at low rates at the single‐cell level, so that probabilistic models carry a high risk of false negatives for sparse ligand–receptor pairs. On the other hand, the probability scoring of such methods is mathematically biased towards highly abundant, broadly expressed pathways (such as SPP1 and MIF), systematically diluting low‐abundance chemokine signals, which also explains why pathways such as SPP1 dominate our results.

Notably, in Supporting Information Figure 5E, the MIF–(CD74 + CXCR4) interaction was detected only in the direction from ACKR1^+^ ECs to B cells, cDC2 and pDCs, and not towards T cells. This suggests that although *CXCR4* is detectable in the present data, its detection level within the T‐cell population is insufficient to support a significance determination by the probabilistic model; at the same time, MIF, as a highly abundant and broadly acting ligand, more readily passes the probability threshold, whereas the low‐abundance *CXCL12* is diluted—together accounting for the absence of the CXCL12–CXCR4 axis in the computational inference. We therefore turned to direct evidence at the spatial and protein levels to validate this axis.

In the spatial transcriptomic data of human carotid atherosclerotic plaques, we quantified the Euclidean distance from each CD8^+^ T‐cell spot to the nearest ACKR1^+^ EC spot, normalised against an equal number of randomly sampled spots within the same sample (Figure 9A–C). The results showed that, relative to a random distribution, CD8^+^ T cells were significantly spatially enriched in the vicinity of ACKR1^+^ ECs (Wilcoxon rank‐sum test, *p* = 3.99e−04), providing direct spatial evidence for ‘local in situ recruitment’.

**FIGURE 9:**
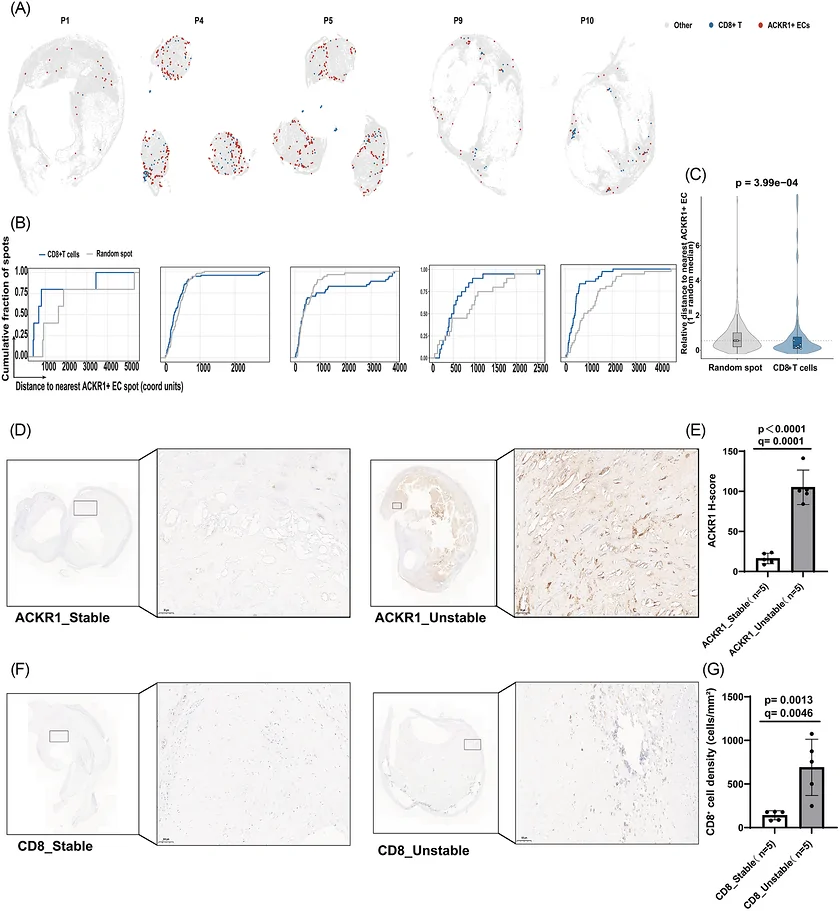
Validation by spatial transcriptomics and in situ immunohistochemistry. (A) Scatter plots showing the spatial distribution of ACKR1^+^ endothelial cell (EC) spots (red) and CD8^+^ T‐cell spots (blue) in five plaque sections (P1, P4, P5, P9 and P10); the remaining spots are shown in grey. (B) Cumulative distribution curves of the distance from CD8^+^ T‐cell spots (blue) and random control spots (grey) to the nearest ACKR1^+^ EC spot in each sample; panels are arranged in the same order as in A. (C) Violin–box plots of the relative distances of CD8^+^ T‐cell spots and random control spots after pooling all five samples (normalised to the median of the random control spots in each sample; 1 = random median; Wilcoxon rank‐sum test, *p* = 3.99e−04). (D) Representative images of atypical chemokine receptor 1 (ACKR1) immunohistochemical staining in stable and unstable plaques; left, whole‐slide scans; right, magnified views of the boxed regions (scale bar = 50 µm). (E) Comparison of ACKR1 H‐scores between groups (stable, *n* = 5; unstable, *n* = 5). (F) Representative images of CD8 immunohistochemical staining (scale bar = 100 µm). (G) Comparison of CD8^+^ cell density (cells/mm^2^) between groups. In E and G, data are presented as the mean ± standard deviation (SD), with dots representing individual measured values; values were log_10_‐transformed and compared using two‐tailed independent‐samples *t*‐tests, and *q* denotes the Benjamini–Hochberg‐adjusted *p* value.

At the protein level, we validated the key molecules in situ by IHC. The ACKR1‐positive signal was localised principally to the endothelium of intraplaque neovessels, and its expression was significantly higher in unstable than in stable plaques (H‐score: 105.08 ± 21.47 vs. 16.29 ± 6.55; GMR = 6.82; 95% CI, 4.21–11.05; *q* = 1.13e−04, Cohen's *d* = 5.80; Figure 9D,E). The infiltration density of CD8^+^ T cells was likewise significantly increased (690.47 ± 322.62 vs. 141.47 ± 51.80 cells/mm^2^; GMR = 4.63; 95% CI, 2.23–9.64; *q* = .0046, Cohen's *d* = 3.05), with positive cells distributed mostly in regions rich in neovessels (Figure 9F,G). Taken together with the spatial proximity analysis, the in situ protein expression and the population‐level transcriptional correlations described above (Figures 5F and 6A–C), these multiple orthogonal lines of evidence jointly support an association between ACKR1^+^ ECs and the local enrichment of CD8^+^ T cells.

We further quantified the model feature molecules and key nodes of the mechanistic chain by IHC (Figure 10A–F). Consistent in direction with the transcriptomic and proteomic results, the lactate efflux transporter SLC16A3/MCT4 (H‐score: 19.21 ± 15.52 vs. 4.62 ± 3.92; GMR = 4.74; 95% CI, 1.38–16.25; *q* = .0337) and HIF‐1α (17.45 ± 9.01 vs. 5.67 ± 5.43; GMR = 4.02; 95% CI, 1.27–12.77; *q* = .0337) were expressed at significantly higher levels in unstable than in stable plaques. SLC16A4/MCT5 showed the opposite direction, being decreased in unstable plaques (23.62 ± 13.63 vs. 58.31 ± 26.43; GMR = .35; 95% CI,  .12–.99), but did not reach statistical significance after multiple testing correction (unadjusted *p* = .0475, *q* = .0554) and is therefore reported only as a trend. This direction is consistent with the sign of its coefficient as a protective variable in the model, and also accords with the interpretation that it derives principally from SMCs and declines with degeneration of the fibrous cap structure.

**FIGURE 10:**
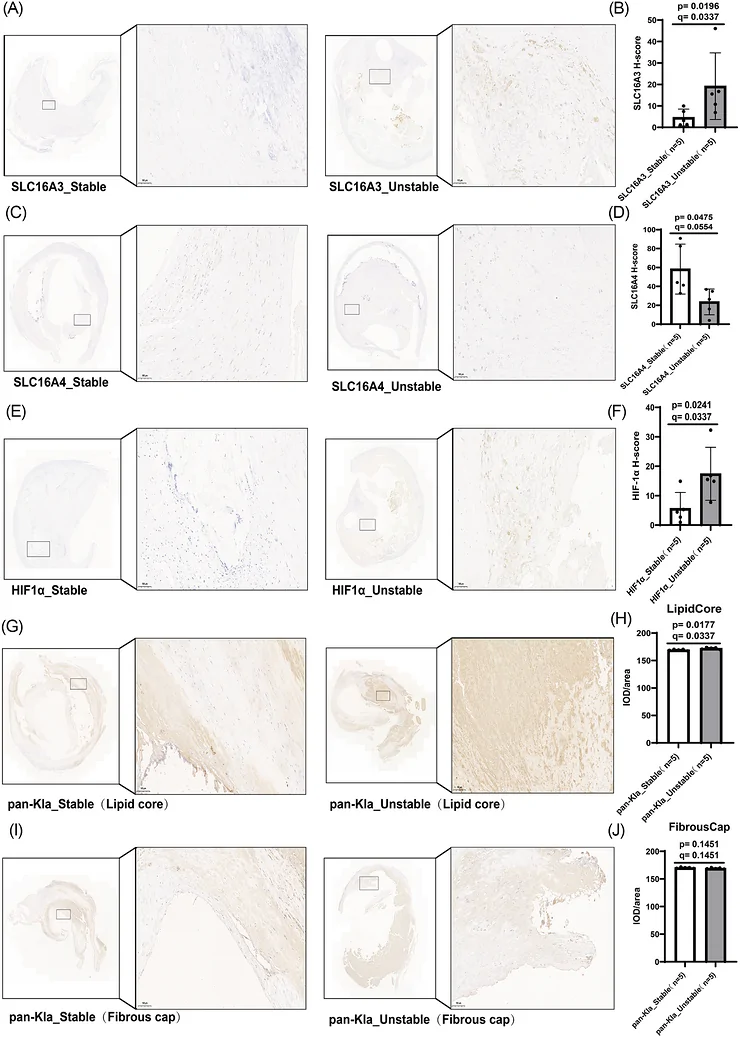
In situ immunohistochemical quantification of model signature molecules and lactylation. (A, C, E) Representative immunohistochemical images of SLC16A3/monocarboxylate transporter 4 (MCT4) (A), SLC16A4/MCT5 (C) and HIF‐1α (E) in stable and unstable plaques; left, whole‐slide scans; right, magnified views of the boxed regions (scale bar = 100 µm). (B, D, F) Comparison of H‐scores for the corresponding markers between groups (stable, *n* = 5; unstable, *n* = 5). (G, I) Representative images of pan‐lysine lactylation (Pan‐Kla) staining in the lipid core region (G) and the fibrous cap region (I) (scale bar = 100 µm). (H, J) Comparison of the mean optical density (IOD/Area) of Pan‐Kla staining in the lipid core region (H) and the fibrous cap region (J) between groups. Data are presented as the mean ± standard deviation (SD), with dots representing individual measured values. H‐scores were log_10_‐transformed before analysis, whereas Pan‐Kla optical density values were analysed on the original scale; all comparisons were performed using two‐tailed independent‐samples *t*‐tests, and *q* denotes the Benjamini–Hochberg‐adjusted *p* value (adjusted jointly across the seven molecular markers). Although SLC16A4 showed a decreasing trend in unstable plaques, the difference did not reach statistical significance after adjustment (*q* = .0554).

In addition, Pan‐Kla staining was quantified separately in the two anatomical compartments of the lipid core and the fibrous cap (Figure 10G–J). In the lipid core region, the overall lactylation level of unstable plaques was higher than that of stable plaques (IOD/Area: 172.07 ± 1.74 vs. 169.25 ± 1.22; *q* = .0337), whereas in the fibrous cap region no significant difference was observed between groups (169.15 ± 1.15 vs. 170.28 ± 1.06; *q* = .1451). This regional specificity suggests that the elevation of intraplaque lactylation occurs mainly in the lipid core region, consistent with the hypoxic and highly glycolytic microenvironment of that region, and corroborates the elevated glycolysis and lactylation module scores of ACKR1^+^ ECs at the single‐cell level (Figure 3D,E).

It must be stated candidly that the absolute magnitude of this difference is small (GMR = 1.017; 95% CI, 1.004–1.030, i.e., approximately 1.7%) and that IOD/Area values are concentrated within the narrow interval of 167–174; its statistical significance derives in part from the very small within‐group variability (SDs 1.2–1.7). This result should therefore be understood as directionally supportive evidence rather than as a strong effect, and requires confirmation by more direct in situ measurement of lactate concentration.

In summary, the association between ACKR1^+^ ECs and CD8^+^ T‐cell recruitment and plaque vulnerability is jointly supported by differential expression and correlations in the bulk cohorts, feature selection by an independent predictive model, quantitative differences in infiltration at the protein level, and spatial proximity analysis. The difference in cell proportion at the single‐cell level did not reach significance owing to the limited sample size and serves only as trend‐level evidence; the IHC cohort likewise comprised only five cases per group, and the related results are similarly exploratory in nature.

## 4 DISCUSSION

The rupture of atherosclerotic plaques and subsequent thrombosis constitute the core pathological foundation of fatal cardiovascular events such as acute myocardial infarction and ischaemic stroke.^1^, ^22^ In the current era of precision medicine, the early identification of vulnerable plaques with a high risk of rupture and the elucidation of their underlying driving mechanisms represent a major challenge for targeted cardiovascular intervention.^23^ By integrating large‐scale scRNA‐seq, bulk RNA‐seq, and proteomic data, this study dissected the ‘endothelial–immune–metabolic’ reprogramming within the plaque microenvironment. We not only identified a specific ACKR1^+^ EC subpopulation closely associated with intraplaque neovascularisation but also proposed a hypothetical pathway that may be initiated by glycolysis and intracellular lactate accumulation, proceed through altered HIF‐1α stability to upregulate *CXCL12*, and ultimately recruit CD8^+^ T cells. More importantly, using this multi‐omics mechanistic network as a theoretical foundation, we applied ML algorithms such as LASSO penalised regression to develop and validate a four‐feature lactate–immune risk score model, which retained discriminative ability at both the lesion‐progression stratification and disease‐identification levels and across three vascular beds (carotid, femoral and infrapopliteal arteries). This study provides an interpretable computational framework for the molecular classification of vulnerable plaques and offers candidate targets for the future development of non‐invasive risk assessment. The overall findings of this study are summarised in Figure 11.

**FIGURE 11:**
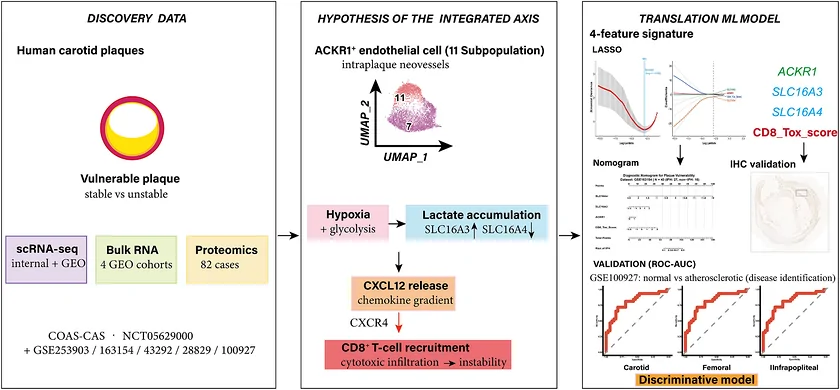
Graphical abstract: An ACKR1^+^ endothelial lactate–immune axis and a four‐feature discriminative signature. Left (discovery data): Human carotid plaques from the Carotid Artery Stenosis Study (COAS‐CAS) cohort (NCT05629000), classified histologically as stable or unstable, were analysed by scRNA‐seq (in‐house and Gene Expression Omnibus [GEO]), bulk transcriptomics (four GEO cohorts: GSE163154, GSE43292, GSE28829 and GSE100927) and proteomics (82 cases). Middle (hypothesis of the integrated axis): ACKR1^+^ ECs (Cluster 11) correspond to intraplaque neovessels and are transcriptionally distinct from ACKR1^−^ ECs (Cluster 7). Within this subpopulation, hypoxia and enhanced glycolysis are associated with intracellular lactate accumulation and an imbalance of the lactate transporter network (*SLC16A3* upregulated, *SLC16A4* downregulated), which is in turn linked to CXCL12 release and to the recruitment of CXCR4^+^ CD8^+^ T cells and plaque instability. This axis is an association‐based hypothesis derived from multi‐omics and in situ evidence; its causality remains to be confirmed by functional experiments. Right (translation): Least absolute shrinkage and selection operator (LASSO) regression selected a four‐feature signature (*ACKR1*, *SLC16A3*, *SLC16A4* and the CD8_Tox_Score), presented as a nomogram and validated in situ by immunohistochemistry. Receiver operating characteristic (ROC) curves show the performance of the locked model in the carotid, femoral and infrapopliteal arteries of GSE100927, in which samples are grouped as normal versus atherosclerotic tissue; these curves therefore reflect disease identification rather than the discrimination of plaque vulnerability.

The high heterogeneity of ECs in the development and progression of atherosclerosis has been a research hotspot in single‐cell omics in recent years.^24^ The ACKR1^+^ EC subpopulation identified here at single‐cell resolution exhibits high transcriptional specificity and is highly co‐expressed with markers of intraplaque neovascularisation. Traditional vascular biology posits that plaque ECs primarily drive early leukocyte rolling and adhesion through the expression of classical adhesion molecules.^25^ However, we observed a scientifically noteworthy anomaly: classical acute inflammatory signalling pathways were paradoxically downregulated in this ACKR1^+^ EC subpopulation.

Although *HIF1A* transcript levels showed an increasing trend in this subpopulation, severe hypoxic stress would, according to classical theory, activate the NF‐κB pathway and lead to a synchronous elevation of pro‐inflammatory cytokines such as TNFα accompanied by widespread inflammatory infiltration.^26^, ^27^ Surprisingly, the TNFα and NF‐κB signalling pathways in ACKR1^+^ ECs were significantly downregulated. This phenotype is directly supported by transcriptomic evidence in the present study: GSEA of the in‐house single‐cell data showed significant downregulation of the TNFA SIGNALLING VIA NFKB pathway (Figure 3A), and KEGG analysis likewise indicated overall downregulation of inflammatory and fluid shear stress‐related pathways (Figure 2H); *CXCL12* was also expressed in this subpopulation, although it is derived from multiple cell types within the plaque (Table S6). This coexisting ‘low‐inflammation, high‐chemotaxis’ phenotype was independently reproduced in the external single‐cell cohort GSE253903 (Supporting Information Figure 1C,F,H). This indicates that these cells are not merely in a state of ‘acute hypoxic stress’ but have entered a metabolically remodelled homeostasis. This paradoxical phenotype can be reasonably explained by local lactate metabolism. Multiple studies have confirmed that high concentrations of lactate accumulating in the microenvironment or intracellularly can act as a key negative regulatory signal, potently blocking the conventional pro‐inflammatory cascade by directly inhibiting NF‐κB activation and nuclear translocation.^28^, ^29^ As a decoy receptor lacking classical G protein‐coupled receptor signalling capacity, the core function of ACKR1 is not to mediate inflammatory cascades but to bind, internalise and transcytose chemokines, thereby establishing chemotactic gradients.^30^, ^31^ A recent breakthrough study published in *Circulation* also indicated that ACKR1^+^ plaque neovessels can serve as specific conduits that potently mediate CD8^+^ T‐cell infiltration by secreting and transporting CXCL12.^10^ Taken together, the downregulation of classical acute inflammatory pathways is very likely a phenotypic tolerance mechanism adopted by ACKR1^+^ ECs in a lactate‐rich microenvironment to avoid early endothelial apoptosis caused by excessive inflammation. This state enables them to act as a persistent local immune hub that continuously delivers destructive effector T cells to the lipid core of the plaque.

Although the specific recruitment of CD8^+^ T cells by ACKR1^+^ endothelium has been established, the microenvironmental stress that triggers this endothelial phenotypic switch has remained unresolved. This study provides an answer from a metabolic perspective: the abnormal immune recruitment function of ACKR1^+^ ECs may be driven by their hyperactive glycolysis and the resulting abnormal intra‐ and extracellular lactate dynamics.

As the lipid core of the plaque expands, a severe hypoxic microenvironment forms in the deeper layers. To maintain survival and angiogenesis, ECs are forced to undergo metabolic reprogramming. This process is highly consistent with the marked activation of the glycolysis‐related TF *GLI2* observed in our study. EC activation and microangiogenesis depend heavily on metabolic rewiring towards glycolysis, particularly the upregulation of the key enzymes *PFKFB3* and *HK2*.^32^ Extensive previous studies have confirmed that microenvironmental signals can activate the Hedgehog/GLI pathway, promoting nuclear translocation of GLI2, which directly binds the promoter regions of rate‐limiting glycolytic enzymes and thereby strongly drives cellular glycolysis.^33^, ^34^ This switch leads directly to the rapid intracellular accumulation of the metabolic byproduct lactate.

Regarding the cellular source of intraplaque CXCL12, our single‐cell data present a picture that warrants cautious interpretation. *CXCL12* showed a multicellular expression pattern within the plaque: the proportion of positive cells was highest in SMCs (57.9% vs. 34.3%), followed by certain macrophage subpopulations (32.6%), with ACKR1^+^ ECs at 21.2%, ranking fourth among all 18 cell subpopulations (Table S6). This distribution is consistent with a previous single‐cell study of human plaques, which reported that fibroblast‐like SMCs participate in intraplaque lymphocyte recruitment by secreting chemokines such as CXCL12 and CCL19.^14^ We therefore do not consider ACKR1^+^ ECs to be the principal producers of CXCL12 within the plaque; only when the comparison is restricted to ECs is their positive rate higher than that of ACKR1^−^ ECs (21.2% vs. 13.1%).

On this premise, the role of ACKR1^+^ ECs in the mechanistic hypothesis proposed here needs to be redefined. What distinguishes this subpopulation from other cell types is neither its metabolic activity nor its chemokine output, but rather its identity as neovascular endothelium: the positive rates of *ACKR1* and *PLVAP* in this subpopulation were 51.1% and 52.9%, respectively, the highest among the 18 subpopulations (Table S6). Intraplaque neovessels constitute the physical interface through which circulating immune cells enter the plaque parenchyma, and the permeability and adhesion molecule expression of their walls determine whether immune cells can extravasate and be retained. Our spatial transcriptomic analysis showed that CD8^+^ T cells were significantly spatially adjacent to ACKR1^+^ ECs (*p* = 3.99e−04), and IHC showed that ACKR1 and CD8 were concordantly elevated in unstable plaques, with positive signals distributed in adjacent regions. Combined with the finding of Parma et al. that CD8^+^ T‐cell recruitment was markedly reduced after CXCR4 antagonist blockade in an ex vivo human plaque culture model,^10^ we are inclined to regard ACKR1^+^ neovessels as a structural portal in this process rather than the principal source of the chemotactic signal—the local CXCL12 gradient may be shaped jointly by multiple cell types, including SMCs, whereas the neovessels determine where immune cells can traverse the vessel wall and enter the plaque.

As for the upstream regulation of *CXCL12* expression by the ECs themselves, this study can provide only indirect clues. In ACKR1^+^ ECs, the activities of the two classical pathways previously reported to induce endothelial *CXCL12*—the fluid shear stress pathway and the ox‐LDL/oxidative stress response—were both low,^35^, ^36^ suggesting that *CXCL12* expression in this subpopulation may be regulated by other factors. Given the hypoxic and glycolytic microenvironment in which this subpopulation resides, one possible explanation involves HIF‐1α: cell line studies have reported that intracellular lactate accumulation can induce lactylation of HIF‐1α, a modification that interferes with VHL recognition of HIF‐1α and thereby slows its ubiquitin‐mediated degradation, while also promoting HIF‐1α accumulation under the catalysis of EP300^12^, ^37^; HIF‐1α can in turn bind directly to the *CXCL12* promoter region and upregulate its transcription.^38^, ^39^ Consistent with this, we observed reduced expression of *EGLN1* (*PHD2*), the key rate‐limiting enzyme of the HIF‐1α degradation pathway, in ACKR1^+^ ECs (avg log_2_FC = −.221, adjusted *p* = 4.95e−07), and single‐label IHC confirmed that HIF‐1α protein expression was significantly higher in unstable than in stable plaques (H‐score: 17.45 ± 9.01 vs. 5.67 ± 5.43; GMR = 4.02; 95% CI, 1.27–12.77; *q* = .0337, Cohen's *d* = 1.76). It should be stated explicitly that the specific molecular event of lactylation‐mediated HIF‐1α stabilisation was not directly verified in this study, and the above inference rests on previous cell line work; what this study can objectively demonstrate is a set of mutually corroborating associations: enrichment of HIF‐1α protein in regions of ACKR1^+^ ECs, reduced *EGLN1* expression and a relative increase in *CXCL12* within the endothelial compartment. Confirmation of this step will require direct experiments such as co‐immunoprecipitation, site‐specific lactylation antibodies and mass spectrometry.

In addition, our ML model accurately selected the lactate metabolism‐related monocarboxylate transporters *SLC16A3* (MCT4) and *SLC16A4* (MCT5).^40^, ^41^ The two showed opposite directions of expression change in lesions. This constitutes a pathologically self‐consistent inference: hyperactive glycolysis in ECs under hypoxia can lead to intracellular lactate accumulation; based on the mechanism reported in previous cell line studies whereby lactylation enhances HIF‐1α stability, this process may be further associated with upregulated *CXCL12* transcription; subsequently, to maintain intracellular acid–base homeostasis, ECs compensatorily upregulate the efflux transporter *SLC16A3* to export excess lactate, and the acidified extracellular microenvironment may further reshape local immune homeostasis. It should be noted that the argument for intracellular lactate accumulation in this study rests on the general upregulation of core glycolytic enzymes and the significant upregulation of the efflux transporter *SLC16A3*, and does not depend on the transport direction of *SLC16A4*. Given that the substrate specificity and transport direction of MCT5 (*SLC16A4*) remain contested in the literature, we do not assign it a definite transport mechanism but position it as a molecular readout negatively correlated with lesion progression: the aberrant upregulation of the efflux transporter *SLC16A3* accompanied by the significant downregulation of *SLC16A4* reflects an imbalance in the expression of the lactate transporter network and, collectively, lactate metabolic dysregulation in the pathological microenvironment.

Driven continuously by the aforementioned metabolic–endothelial communication axis, large numbers of CD8^+^ T cells are specifically recruited into the plaque. Traditional theories focused heavily on macrophages; however, high‐resolution scRNA‐seq studies have clearly revised this view, showing that T cells—particularly cytotoxic CD8^+^ T cells—are the predominant pro‐inflammatory lymphocyte population in the human plaque microenvironment.^9^, ^42^ In our validation cohorts, we clearly observed that the CD8^+^ T‐cell infiltration score was significantly elevated in patients with IPH and was positively correlated with *ACKR1* and lactate metabolism markers.

During the progression of vulnerable plaques, CD8^+^ T cells play an extremely destructive role. Activated CD8^+^ T cells release substantial amounts of perforin and granzyme B into the extracellular space. Immunopathological studies have confirmed that granzyme B can directly induce widespread apoptosis of SMCs in the fibrous cap, leading to rapid cap thinning^43^; concurrently, the IFN‐γ they secrete further inhibits the capacity of SMCs to synthesise new collagen.^44^ Furthermore, apoptosis of plaque neovessel ECs under attack by cytotoxic factors directly disrupts the physical integrity of the microvessels, causing massive erythrocyte leakage and thereby triggering IPH and rapid expansion of the lipid necrotic core.^45^ Therefore, CD8^+^ T cells recruited via the ACKR1/CXCL12 axis may represent a key link connecting upstream metabolic dysregulation to the ultimate physical rupture of the plaque.

It should be noted that this study did not perform functional blockade experiments of the CXCL12–CXCR4 axis; the above discussion of the recruitment role of this axis is an inference based on the spatial proximity, IHC, and cohort correlation evidence of the present study, combined with previous functional research. A recent study published in *Circulation* has confirmed the role of this axis in mediating CD8^+^ T‐cell recruitment in an ex vivo human plaque culture model, using the CXCR4 antagonist AMD‐3100 and pertussis toxin blockade.^10^ We cite this independent evidence as corroboration of the biological plausibility of this axis while making clear that the functional conclusion derives from previous work; the contribution of the present study lies in establishing an association between this axis and plaque metabolic reprogramming and vulnerability at the transcriptional, protein and spatial levels in human cohorts.

It should further be noted that the above associations derive mainly from bulk tissue transcriptomes and may be partly influenced by differences in cell composition in unstable plaques. We therefore performed cell composition adjustment analyses. The results showed that the correlations between *ACKR1* and some glycolytic and lactate transport molecules (e.g., *HK2*, *SLC16A3* and *SLC16A4*) were markedly attenuated after adjustment for cell proportions (adjusted partial correlations *r* = .03,  .20 and  .03, respectively; all non‐significant), suggesting that these associations largely reflect the overall increase in the abundance of ACKR1^+^ endothelium (neovessels) and immune cells in unstable plaques—that is, covariation at the level of cell abundance rather than strict intracellular co‐regulation; this is itself consistent with the pathological feature of neovascular proliferation in vulnerable plaques. By contrast, the HIF‐1α–CXCL12 relationship at the core of the mechanistic chain remained robust after adjustment for cell composition (partial correlation *r* = .55, *p* = .0003), indicating that it operates at the intracellular regulatory level. Accordingly, we define the phenomena revealed in this study precisely as two superimposed but equally real levels: the enrichment of ACKR1^+^ ECs in unstable plaques (cell composition level) and the HIF‐1α–CXCL12‐centred regulatory coupling within these cells (intracellular level). This renders the mechanistic interpretation more prudent and avoids attributing all bulk correlations to intracellular co‐regulation.

Based on this mechanistic framework, we employed the LASSO penalised regression algorithm to perform rigorous feature reduction within this large network. Ultimately, we identified core features encompassing *ACKR1*, *SLC16A3*, *SLC16A4* and the CD8_Tox_Score, and constructed a LR diagnostic model. In cardiovascular artificial intelligence, such a feature selection strategy driven by clearly defined pathological mechanisms has been shown to confer biological robustness and clinical interpretability far exceeding those of purely black‐box data mining.^46^, ^47^

This diagnostic model performed excellently not only in rigorous internal LOOCV but also demonstrated outstanding generalisability across multiple independent external cohorts. Specifically, we conducted comprehensive blind testing in three independent cohorts. The GSE43292 cohort contained paired samples of advanced high‐risk carotid plaques and adjacent early‐stage tissues; GSE28829 covered early and advanced carotid plaques; and GSE100927 was a large cohort encompassing not only carotid arteries but also peripheral vascular beds such as the femoral and infrapopliteal arteries. Because the initial diagnostic model was trained strictly on the pathological stability of carotid plaques (IPH vs. non‐IPH), the grouping criteria of these three validation cohorts are not clinicopathologically equivalent to the classical binary classification of ‘vulnerable versus stable’. Despite this objective baseline heterogeneity, the model maintained a high AUC, which conveys a deeper biological signal: the core features extracted in this study do not change only at the moment of plaque rupture but precisely and dynamically capture the entire pathological trajectory of atherosclerosis from early lipid deposition to advanced high‐risk lesions.

Furthermore, the excellent performance of the model in the GSE100927 cohort, which includes lower extremity peripheral vessels, further supports the concept of ‘polyvascular disease’ that is currently attracting considerable international attention.^48^ It should be noted that the grouping in this cohort was normal versus diseased tissue; what is supported here is therefore the consistency of the signature in identifying atherosclerotic lesions across different vascular beds, rather than its ability to discriminate vulnerability within each vascular bed. This indicates that atherosclerosis across different vascular beds, such as the carotid and peripheral lower extremity arteries, shares a highly unified pathophysiological basis driven by metabolic reprogramming and immune cell recruitment. This multivariable model, which retains discriminative ability across multiple vascular beds, provides a quantifiable tool for the molecular classification of ex vivo plaques; developing it into a clinical instrument for longitudinal monitoring will require validation in prospective cohorts.

This study has several limitations that warrant careful consideration. First, it is fundamentally a retrospective, cross‐sectional observational study, and the molecular associations revealed are insufficient to establish causality; the mechanistic chain we propose—‘metabolic reprogramming of ACKR1^+^ ECs—lactate accumulation—HIF‐1α stabilisation (lactylation)—CXCL12 release—CD8^+^ T‐cell recruitment—plaque vulnerability’—is essentially an inferential hypothesis constructed from multi‐omics associations and previous literature. In particular, the key molecular event of HIF‐1α lactylation was not directly verified experimentally in this study but was inferred from previous basic research in cell lines; moreover, the highly glycolytic/hypoxic metabolism within the plaque is not unique to ACKR1^+^ ECs but is a regional microenvironmental feature shaped jointly by multiple cell types, including macrophages. These causal relationships and key molecular nodes will require confirmation through rigorous in vitro and in vivo functional experiments (e.g., lactate intervention in ECs, site‐specific detection of HIF‐1α lactylation, CXCR4 blockade and gene‐edited animal models). In addition, although this study confirmed the in situ spatial proximity of ACKR1^+^ ECs and CD8^+^ T cells by spatial transcriptomics, the intraplaque lactate concentration gradient itself was not directly measured, and this gap remains to be filled at the in situ tissue level by technologies such as spatial metabolomics.

Second, the number of unstable samples in the single‐cell analysis was small (*N* = 3), resulting in insufficient statistical power for between‐group comparisons at the single‐cell level, particularly for differences in cell proportions; the related results (such as the increasing trend in the proportion of cytotoxic CD8^+^ T cells) should be regarded only as exploratory findings, and the core conclusions of this study rest primarily on the larger bulk transcriptomic cohorts. Furthermore, some correlations based on bulk tissue may be influenced by differences in cell composition: cell composition adjustment analysis showed that the correlations of *ACKR1* with certain glycolytic/lactate transport molecules were attenuated after adjustment, suggesting that they partly reflect the overall enrichment of ACKR1^+^ neovessels and immune cells in unstable plaques, whereas the core HIF‐1α–CXCL12 relationship remained robust after adjustment. It should also be noted that the conclusions regarding CD8^+^ T cells in this study are based on the expression levels of cytotoxic effector molecules rather than on cell proportions. Between‐group comparisons of CD8^+^ T‐cell proportions did not reach significance in either independent single‐cell cohort, which reflects both the limited sample sizes and differences in sampling sites of the single‐cell cohorts and the fact that cell number and activation state represent two distinct facets of immune infiltration. Validation with cell proportion as the endpoint remains to be undertaken in larger single‐cell or flow cytometry cohorts.

Third, this study has two limitations at the level of clinical variables. First, the public transcriptomic cohorts used for training and external validation lacked individual clinical annotations suitable for multivariable adjustment, so the independence of the locked model from clinical factors could only be argued indirectly through the within‐patient paired design of GSE43292. Although the in‐house proteomic cohort had complete clinical annotation and permitted direct adjustment, its measurements were at the protein rather than the transcript level, and ACKR1, SLC16A4 and the CD8 cytotoxic molecules were not stably detected by DIA mass spectrometry; moreover, this cohort lacked records of statin and antihypertensive medication use, and symptomatic status was represented only by the indirect proxy of a history of cerebral infarction/TIA. Its adjustment results therefore apply to the protein‐level conclusions regarding glycolysis and cannot be equated with clinical adjustment of the four‐feature model. Accordingly, ‘excluding confounding’ and ‘quantifying incremental value’ remain two distinct propositions: the present study has accomplished only the former, and the incremental value of this signature relative to established clinical risk factors cannot yet be quantified; we therefore position it as a complement to, rather than a replacement for, established clinical risk factors. Second, with a limited training set sample size (*N* = 43), the precision of the calibration estimates was constrained—the calibration slope under nested LOOCV was 1.105 (95% CI,  .693–2.570), and the Hosmer–Lemeshow test was underpowered—so the calibration results support only the weaker conclusion that ‘no evident overconfidence was observed’ and are insufficient to claim accurate calibration. In addition, this study lacked paired normal/healthy vascular control tissue and therefore could not directly compare the abundance of ACKR1^+^ ECs between healthy and diseased vessels within the present data; available evidence suggests that ACKR1^+^ ECs correspond to venous/venular‐type endothelium that exists under physiological conditions, and their enrichment in plaques derives mainly from the proliferation of intraplaque neovessels. The precise substrate specificity and transport direction of SLC16A4 (MCT5) in ECs also remain unclear; this study includes it in the model only as a molecular marker negatively correlated with lesion progression, and its biological function awaits elucidation by subsequent transport experiments. Furthermore, because feature standardisation in this model is referenced to the expression distribution within each cohort, its output is a relative risk score rather than an absolute probability and thus requires a reference population; it cannot currently be applied directly to the interpretation of a single isolated sample. Applying it to individualised clinical decision‐making would require the determination of fixed standardisation parameters and decision thresholds in prospective cohorts.

Fourth, different grouping criteria were used across the datasets in this study (histopathological features for the in‐house cohort and GSE163154, patient symptoms for GSE253903 and disease identification of normal vs. plaque tissue for GSE100927). Although all of these criteria relate to plaque progression/vulnerability, they are not equivalent to one another, nor are any of them based on clinical outcomes confirmed by prospective follow‐up. Moreover, the available data of this study alone cannot confirm that the excised plaques were the culprit lesions responsible for clinical events. The performance of the model under each endpoint should therefore be interpreted with caution in light of the specific pathological reference standard, and its strict predictive value for plaque rupture risk remains to be validated. In addition, all samples analysed in this study were atherosclerotic plaques excised by CEA, without paired normal vascular controls, so the abundance of ACKR1^+^ ECs in healthy versus diseased vessels could not be directly compared at single‐cell resolution. On this question, the external cohort GSE100927 (normal arteries vs. atherosclerotic lesions) provides partial insight: *ACKR1* did not differ significantly between the two groups, consistent with its property as a constitutive marker of venous‐type endothelium and indicating that this cell population is not lesion‐specific; by contrast, *ACKR1* was significantly elevated in the comparison of IPH versus non‐IPH within plaques, suggesting an association with lesion progression. However, these are indirect, bulk‐level findings that cannot resolve cell abundance from cell state, and rigorous validation will require single‐cell studies that include normal vascular controls.

Fifth, the risk assessment model constructed in this study takes as its input the molecular features of ex vivo plaque tissue, which can be obtained only after excision by CEA; the model therefore currently has no direct clinical value for preoperative non‐invasive prediction. It is presently positioned as a research tool for mechanistic dissection and the molecular classification of ex vivo plaques, and as a source of candidate targets for future non‐invasive translation. Developing it into a clinically applicable non‐invasive risk assessment method (e.g., based on imaging or peripheral blood markers) will require prospective cohorts with complete clinical annotation, strictly matched pathological grouping, and contemporaneously collected tissue transcriptomes. On this basis, further evaluation of the feasibility of translating this signature into targeted molecular imaging probes (e.g., for PET‐CT) represents another possible route towards preoperative non‐invasive assessment. At the circulating level, the most discriminative components of this signature axis—the CD8^+^ T‐cell cytotoxic effector molecules (granzyme B, perforin and IFN‐γ)—can in principle be measured in peripheral blood and thus hold the greatest potential for non‐invasive translation; erythrocyte‐surface ACKR1 (the Duffy antigen), which acts as a chemokine reservoir influencing plasma chemokine concentrations, offers another indirect route worth evaluating. However, there is as yet no evidence for a quantitative relationship between these circulating indicators and intraplaque molecular features, which will need to be established in prospective cohorts with contemporaneous collection of plaque tissue and paired peripheral blood.

## 5 CONCLUSIONS

Through a multidimensional integrated analysis of scRNA‐seq, bulk RNA‐seq and proteomics, this study confirms the possible existence of a metabolic reprogramming‐driven ‘endothelial–immune’ pathogenic axis in carotid atherosclerotic lesions. Our findings suggest that ACKR1^+^ ECs under hypoxic conditions display an actively glycolytic phenotype accompanied by molecular features of lactate accumulation; in light of the mechanism reported in previous cell line studies whereby lactylation enhances HIF‐1α stability, this state may be associated with upregulated *CXCL12* expression and with the enrichment of cytotoxic CD8^+^ T cells within the plaque, and thereby with structural destabilisation of the plaque. It must be emphasised that this mechanistic chain is an inferential hypothesis constructed from multi‐omics associations and corroborating literature, and its causality remains to be confirmed by functional experiments.

Building on this, we used ML algorithms to select and construct a four‐feature molecular discriminant model comprising *ACKR1*, *SLC16A3*, *SLC16A4* and the CD8^+^ T‐cell cytotoxicity score. This model demonstrated good robustness across multiple independent external cohorts spanning different platforms and vascular beds. It should be noted that the grouping criteria of the validation cohorts were not uniform: cohorts stratified by lesion progression directly support the proposition of vulnerability stratification, whereas cohorts grouped as normal versus diseased tissue reflect the disease‐identification capacity of this signature. Together, these results suggest that this ‘lactate–immune’ signature may capture the continuous pathological trajectory from early lipid deposition to advanced vulnerable lesions.

## AUTHOR CONTRIBUTIONS

All the authors contributed to the conception, design, execution and analysis of the study. **Ji Zhang** conceptualised the study, designed the research protocol, performed the multi‐omics data integration and machine‐learning model construction, carried out the statistical analysis and wrote the original draft. **Qijian Zhao, Yufei Wu and Keqiang Shu** refined the study design, performed the histopathological classification and immunohistochemical experiments, participated in data collection and validation and were responsible for data visualisation. **Junye Chen, Kang Li, Yuyao Feng and Xu Zhang** contributed to sample and clinical data collection, data curation and manuscript revision. **Wenjian Liu** provided methodological guidance on the machine‐learning analysis and revised the manuscript. **Zhichao Lai** critically revised the manuscript, supervised the experimental work and contributed to funding acquisition. **Bao Liu** conceived and supervised the overall project, critically revised the manuscript and acquired the primary funding. **Kang Li** also contributed to funding acquisition. **Ji Zhang, Qijian Zhao, Yufei Wu and Keqiang Shu** contributed equally to this work. All the authors read and approved the final manuscript.

## CONFLICT OF INTEREST STATEMENT

The authors declare no conflicts of interest.

**Data generated by our group**. The internal single‐cell and spatial transcriptomes were deposited with our previous study (Lai et al., Nat Cardiovasc Res, 2025) in the Genome Sequence Archive for Human at the National Genomics Data Center under accession HRA006030 (https://ngdc.cncb.ac.cn/gsa‐human), with backups in the China National GeneBank: spatial transcriptomes in the STOmics Database (STT0000052, https://db.cngb.org/stomics/) and single‐cell data in the China National Sequence Archive (CNP0004894, https://db.cngb.org/cnsa/). The plaque proteome is deposited in iProX (PXD062283, https://www.iprox.cn/page/project.html?id=IPX0011499000). The published analysis under that accession grouped samples by American Heart Association lesion type; the 82 samples analysed here are the same data regrouped by histological stability, corresponding to the discovery cohort of Lai et al., J Transl Med 2024;22:247 after exclusion of six erythrocyte‐contaminated samples.

**Processed data**. The processed data required to reproduce the analyses are deposited in Zenodo (https://doi.org/10.5281/zenodo.22141902) and comprise: per‐cell metadata for the internal single‐cell cohort; pseudo‐bulk expression matrices at sample level, at patient level and within ACKR1^+^ ECs; mean expression and percentage of positive cells for all annotation markers in each cluster; the plaque proteome quantification matrix with its stable/unstable group assignment; and the 14 clinical covariates used for confounding adjustment. All direct identifiers (specimen numbers, pathology numbers and dates) have been removed and replaced with sequential anonymous codes; the key linking these codes to the original records is retained by the authors and is not distributed.

**Model coefficients**. The locked model is defined by an intercept of  .4625 and coefficients of −2.6931 (SLC16A4),  .6481 (SLC16A3),  .2556 (ACKR1) and  .7414 (CD8_Tox_Score); these are also shown in Figure 7K. Readers can therefore reproduce the model predictions without retraining.

**Code**. All analysis scripts are available on GitHub (https://github.com/alpinezhang/ACKR1‐lactate‐immune‐signature) and archived in Zenodo (https://doi.org/10.5281/zenodo.22141902). The code covers single‐cell processing and cell‐type annotation, differential expression and pathway enrichment in endothelial subsets, cell‐type deconvolution and partial correlation, pseudo‐bulk analysis, bulk differential expression and enrichment, LASSO feature selection and logistic modelling, fully nested cross‐validation and bootstrap optimism correction, locked‐model external validation, within‐patient paired analysis in GSE43292, and proteome‐wide differential analysis with clinical covariates. The mapping from each script to the corresponding figure is given in the repository README.

No restricted data were used in this study; all analyses can be fully reproduced from the public data and code listed above.

## ETHICS STATEMENT

The Carotid Artery Stenosis Study (COAS‐CAS, Clinical trial: NCT05629000) is a prospective investigation conducted at Peking Union Medical College Hospital (PUMCH). Its aim is to gather atherosclerotic carotid plaques and associated clinical characteristics from patients undergoing carotid endarterectomy (CEA). The study received approval from the Ethics Review Committee of PUMCH (Approval Nos. JS‐2966 and I‐22PJ019), ensuring adherence to ethical guidelines. Prior to participation, written informed consent was acquired from all patients, who also consented to the publication of the research data. This study strictly adhered to the ethical principles outlined in the Declaration of Helsinki.

## CONSENT FOR PUBLICATION

Not applicable.

## Supporting information



Supporting Information

Supporting Information

Supporting Information

Supporting Information

Supporting Information

Supporting Information

Supporting Information

Supporting Information

## Data Availability

**Public datasets**. All public datasets used in this study are available from the Gene Expression Omnibus (https://www.ncbi.nlm.nih.gov/geo/) under accessions GSE163154 (training cohort), GSE43292, GSE28829 and GSE100927 (external validation cohorts) and GSE253903 (external single‐cell cohort).
