# Supplementary material for: Multi‐omics integration and machine learning identify an ACKR1+ endothelial cell‐based lactate‐immune signature associated with carotid plaque vulnerability and progression
Source: Clin Transl Med. 2026 Oct 14;16(10):e70856. doi: 10.1002/ctm2.70856 (PMC13669426; doi:10.1002/ctm2.70856)
Supplement: Supplementary file 1 [file CTM2-16-e70856-s004.docx]

**Multi-omics integration and machine learning identify an ACKR1^+^ endothelial cell-based lactate-immune signature associated with carotid plaque vulnerability and progression**

Ji Zhang^1,#^, Qijian Zhao^1,#^, Yufei Wu^1,#^, Keqiang Shu^1,#^, Junye Chen^1^, Kang Li^1^, Yuyao Feng^1^, Xu Zhang^1^, Wenjian Liu^4,*^, Zhichao Lai^1,*^, Bao Liu^1,2,3,*^

^1^Department of Vascular Surgery, Peking Union Medical College Hospital, Chinese Academy of Medical Sciences and Peking Union Medical College, Beijing, China

^2^Institute of Aging and Health, Chinese Academy of Medical Sciences, Beijing, China.

^3^School of Aging and Health, Peking Union Medical College, Beijing, China.

^4^Faculty of Data Science, City University of Macau, Macau, China.

#These authors contributed equally to this work.

*Correspondence authors.

**Supplementary Fig. 1. Validation based on the external scRNA-seq cohort GSE253903.** Cluster numbering in GSE253903 is independent of that in the in-house cohort and the two are not interchangeable (in-house: ACKR1^+^ ECs, Cluster 11; ACKR1^−^ ECs, Cluster 7). **A.** UMAP plot of the GSE253903 dataset. **B.** Cell type annotation based on canonical markers. **C.** Differential gene expression analysis of ACKR1^+^ ECs (Cluster 7) relative to ACKR1^−^ ECs (Cluster 10). **D.** Identification of specific endothelial cell phenotypes for Cluster 7 (ACKR1^+^ ECs) and Cluster 10 (ACKR1^−^ ECs) based on relevant literature. **E.** Significantly upregulated GO pathways in Cluster 7 (ACKR1^+^ ECs) compared with Cluster 10 (ACKR1^−^ ECs). **F.** Significantly downregulated GO pathways for the same comparison. **G.** Significantly upregulated KEGG pathways for the same comparison. **H.** Significantly downregulated KEGG pathways for the same comparison. **I.** Comparison between Cluster 7 (ACKR1^+^ ECs) and Cluster 10 (ACKR1^−^ ECs) of core enzymes of glycolysis and lactate metabolism, together with *HIF1A* and its prolyl hydroxylase *EGLN1* (*PHD2*). **J.** Comparison of canonical markers and receptors (e.g., *CXCR4*) between Cluster 0 (T cells) and Cluster 2 (CD8^+^ T cells).

**Supplementary Fig. 2. Analysis of IPH and non-IPH samples in the GSE163154 cohort. A.** GO biological process enrichment analysis of significantly upregulated genes in IPH relative to non-IPH samples. **B.** GO biological process enrichment analysis of significantly downregulated genes for the same comparison. **C.** Significantly upregulated KEGG pathways in IPH relative to non-IPH samples. **D.** Significantly downregulated KEGG pathways for the same comparison. **E.** Significantly upregulated HALLMARK pathways in IPH relative to non-IPH samples. **F.** Significantly downregulated HALLMARK pathways for the same comparison. **G.** GSEA showing significant upregulation of the glycolysis pathway in IPH samples. **H.** GSEA showing significant upregulation of the hypoxia pathway in IPH samples.

**Supplementary Fig. 3. External validation of the predictive model across different vascular beds. A.** ROC curve for external validation in the carotid artery of the GSE100927 cohort. **B.** Calibration curve. **C.** DCA curve. **D.** Risk scores. **E.** Comparison of individual variables. **F.** ROC curve for external validation in the femoral artery of the GSE100927 cohort. **G.** Calibration curve. **H.** DCA curve. **I.** Risk scores. **J.** Comparison of individual variables.

**Supplementary Fig. 4. External validation of the predictive model in the infrapopliteal artery. A.** ROC curve for external validation in the infrapopliteal artery of the GSE100927 cohort. **B.** Calibration curve. **C.** DCA curve. **D.** Risk scores. **E.** Comparison of individual variables.

**Supplementary Fig. 5. *ACKR1* expression distribution, glycolysis module scoring, and inferred cell–cell communication of ACKR1^+^ ECs. A.** Violin plot showing the distribution of *ACKR1* expression in ACKR1^−^ ECs (Cluster 7) and ACKR1^+^ ECs (Cluster 11) (avg log_2_FC = 2.37, adjusted *P* = 8.57e-222; proportion of positive cells: 51.1% vs. 13.5%). **B.** *ACKR1* expression projected onto the UMAP of the endothelial subpopulations; color indicates the normalized expression level. **C.** Comparison of module scores based on the HALLMARK_GLYCOLYSIS gene set (Cliff’s δ = −0.204, *P* = 1.71e-35). **D.** Comparison of module scores based on a targeted gene set of glycolytic rate-limiting enzymes (Cliff’s δ = +0.199, *P* = 1.28e-33). **E.** Significant secreted ligand–receptor pairs sent from ACKR1^+^ ECs to cell populations within the plaque, as inferred by *CellChat*; dot size indicates significance and color indicates communication probability. **F.** No significant ligand–receptor pairs from ACKR1^+^ ECs to CD8^+^ T cells (Cluster 4) were detected under the high-sensitivity setting.

**Supplementary Fig. 6. Supplementary internal validation of the model and robustness checks of the principal findings. A.** ROC curve for fully nested LOOCV, together with the frequency with which each candidate feature was independently selected across the 43 folds (*ACKR1*, *SLC16A3*, and *SLC16A4*, 43/43; CD8_Tox_Score, 37/43; all remaining candidates below 12%). **B.** Cell-composition adjustment: correlations among *ACKR1* and glycolytic or lactate-transport genes before and after adjustment for cell type proportions, shown as partial correlation coefficients. The unadjusted correlation coefficients shown in this figure were calculated from the same expression matrix used for the partial correlation analysis (with values averaged across multiple probes) and therefore differ slightly from those in **Fig. 5**, which were derived using the single best probe. **C.** Proportion of CD8^+^ T cells in symptomatic versus asymptomatic patients in the external single-cell dataset GSE253903. **D–F.** Expression of key genes in ACKR1^+^ versus ACKR1^−^ ECs, with multiple testing correction. **G.** Module score of the ACKR1^+^ EC signature in IPH versus non-IPH samples of the GSE163154 cohort. **H.** Metabolic module scores related to glycolysis and lactylation across cell types.

**Supplementary Fig. 7. Control of patient-level clinical confounding and assessment of model calibration. A.** Within-patient paired comparison in GSE43292 (32 patients); gray lines link macroscopically intact tissue (Stary stages I–II) and plaque (Stary stage ≥ IV) from the same patient, with the score computed using the coefficients locked in the training cohort (higher on the plaque side in 28/32 pairs; paired Wilcoxon *P* = 2.93e-05). **B.** Within-patient difference in score (plaque minus intact tissue), sorted by magnitude. **C.** Odds ratios with 95% CIs for each model component from conditional logistic regression stratified by patient (log scale); the interval for *ACKR1* is wide (2.64–324.05) and inference is therefore restricted to direction. **D.** Key proteins from **Fig. 6H** before versus after adjustment for 14 clinical covariates in the in-house proteomic cohort (37 stable, 45 unstable); −log_10_(*P*) on a square-root scale, with the dashed line at *P* = 0.05. **E.** KEGG pathways enriched among upregulated proteins before versus after adjustment (the 10 with the smallest unadjusted *P* among the 13 significant under both settings). **F.** Calibration curve from fully nested LOOCV predictions in GSE163154 (*N* = 43); red line, lowess fit (iter = 0, truncated at the 2nd–98th percentiles of the predicted values); slope estimated by Firth penalized regression. The Brier score here (0.096) is on the nested LOOCV scale and agrees closely with the bootstrap-corrected value in **Fig. 7J** (0.098). **G.** Proteome-wide effect sizes, unadjusted logFC versus logFC adjusted for the same 14 covariates (*r* = 0.994; 1,193 versus 1,011 proteins at *P* < 0.05, 901 shared).

**Supplementary Fig. 8. Between-group comparison of the model features in the training cohort GSE163154. A.** The four model features compared between the IPH (*n* = 27) and non-IPH (*n* = 16) groups; the y axis shows Z-scores standardized within the cohort. **B.** Individual comparison of the five genes comprising the CD8_Tox_Score; the lineage marker *CD8B* did not differ significantly between groups, whereas the cytotoxic effector molecules *GZMB*, *PRF1*, and *GZMA* were significantly elevated. All comparisons used two-sided Wilcoxon rank-sum tests. The proportion of CD8^+^ cytotoxic T cells in the external single-cell cohort is shown in **Supplementary Fig. 6C**.
